# Molecular characterization of Barrett’s esophagus at single-cell resolution

**DOI:** 10.1073/pnas.2113061118

**Published:** 2021-11-18

**Authors:** Georg A. Busslinger, Buys de Barbanson, Rurika Oka, Bas L. A. Weusten, Michiel de Maat, Richard van Hillegersberg, Lodewijk A. A. Brosens, Ruben van Boxtel, Alexander van Oudenaarden, Hans Clevers

**Affiliations:** ^a^Hubrecht Institute, Royal Netherlands Academy of Arts and Sciences, 3584 CT Utrecht, The Netherlands;; ^b^Oncode Institute, Royal Netherlands Academy of Arts and Sciences, 3521 AL Utrecht, The Netherlands;; ^c^Princess Maxima Center for Pediatric Oncology, 3584 CS Utrecht, The Netherlands;; ^d^Department of Gastroenterology and Hepatology, University Medical Center Utrecht, University of Utrecht, 3508 GA Utrecht, The Netherlands;; ^e^Department of Surgical Oncology, University Medical Center Utrecht, University of Utrecht, 3584 CX Utrecht, The Netherlands;; ^f^Department of Pathology, University Medical Center Utrecht, University of Utrecht, 3584 CX Utrecht, The Netherlands

**Keywords:** Barrett’s esophagus, single-cell RNA and DNA analyses, single-base substitution 17 (SBS17a and SBS17b)

## Abstract

Barrett’s esophagus (BE) is categorized, based on morphological appearance, into different stages, which correlate with the risk of developing esophageal adenocarcinoma. More advanced stages are more likely to acquire chromosomal instabilities, but stage-specific markers remain elusive. Here, we performed single-cell DNA-sequencing experiments (scDNAseq) with fresh BE biopsies. Dysplastic BE cells frequently contained chromosomal instability (CIN) regions, and these CIN cells carried mutations corresponding to the COSMIC mutational signature SBS17, which were not present in biopsy-matched chromosomally stable (CS) cells or patient-matched nondiseased control cells. CS cells were predominantly found in nondysplastic BE biopsies. The single-base substitution (SBS) signatures of all CS BE cells analyzed were indistinguishable from those of nondiseased esophageal or gastric cells. Single-cell RNA-sequencing (scRNAseq) experiments with BE biopsies identified two sets of marker genes which facilitate the distinction between columnar BE epithelium and nondysplastic/dysplastic stages. Moreover, histological validation confirmed a correlation between increased CLDN2 expression and the presence of dysplastic BE stages. Our scDNAseq and scRNAseq datasets, which are a useful resource for the community, provide insight into the mutational landscape and gene expression pattern at different stages of BE development.

## Significance

Barrett’s esophagus (BE), the premalignant condition of esophageal adenocarcinoma, is categorized into different stages which correlate with the risk of developing carcinoma. We performed single-cell DNA-sequencing experiments with fresh biopsies, which revealed the appearance of a specific T > C and T > G mutational signature, known as COSMIC signature SBS17, in BE cells that are chromosomally unstable. The SBS17-specific mutations were, however, not detected in chromosomally stable BE cells. Additionally, we performed single-cell RNA sequencing experiments which identified seven genes that facilitate the distinction between different BE stages on histological sections.

Barrett’s esophagus (BE) is the premalignant, benign stage of esophageal adenocarcinoma (EAC), whose incidence rate has increased dramatically over the past decades (1). BE occurs in 1.6 to 11% of Caucasians and is associated with chronic gastroesophageal reflux (2, 3). Morphologically, it is characterized by the epithelial transformation of the healthy multilayered esophageal epithelium to a single-layered columnar one in the distal esophagus (4). A columnar epithelium lacking any signs of intestinal metaplasia (IM) is referred to as gastric-type or columnar (COL) epithelium (5). It is still debated if the presence of IM is a requirement for the identification of BE, highlighted by the different guidelines for BE of the British Society of Gastroenterology and the American College of Gastroenterology (5, 6). Single-layered epithelium containing IM, as characterized by the presence of goblet cells, is associated with increased risk of neoplastic progression (7) and is further categorized as nondysplastic BE (NDBE), BE with low-grade dysplasia (LGD), or BE with high-grade dysplasia (HGD) (8). Some BE patients (<0.5%) progress from NDBE through LGD and HGD stages to EAC (9). However, grading of dysplasia in BE remains a challenge (10, 11). The gold standard is the identification of morphological alterations by hematoxylin/eosin staining (8). Of note, LGD and HGD diagnoses are often down-graded to NDBE after review by expert pathologists (2, 12, 13).

Recent research focused on the identification of BE-specific gene expression patterns. These included, for example, genes commonly detected in the intestine such as *CDX1*, *CDX2*, and *TFF3* (14–16). They are, however, not implemented in clinical practice as they do not reliably distinguish between different BE stages. Recently, a single-cell RNA-sequencing (scRNAseq) study analyzed the cellular composition of NDBE, which identified the expression of *LEFTY1* and *OLFM4* in BE (17). A limitation of this study was its narrow focus on NDBE. Others determined a gene expression signature consisting of 90 genes by microarray analysis to calculate a prediction score for NDBE and HGD distinction (18) or compared the DNA and histone methylation patterns between different stages (19, 20). These approaches are quite labor-intensive and require extensive bioinformatics, which makes them impractical for routine clinical testing. The most useful marker for pathology assessment is the expression of *TP53*, which increases diagnostic accuracy and interobserver agreement between expert pathologists (5, 6, 21).

From a molecular perspective, *CDKN2A* and *TP53* mutations or their epigenetic silencing occur early during BE development and provide a selective growth advantage (22, 23). After the initial selective clonal sweep, additional mutations accumulate during progression, leading to the coexistence of multiple subclones (24, 25). Dysplastic BE stages were correlated with the acquisition of chromosomal instability (CIN), as measured by the loss of heterozygosity of single-nucleotide polymorphisms (SNPs) (26, 27). Low levels of CIN were later confirmed by whole-genome sequencing (WGS) experiments on histological BE sections, whereas CIN increased dramatically in EAC cells (25, 28–30). WGS identified somatic SNP patterns at a genome-wide scale, which revealed an enrichment for the COSMIC single-base substitution signature 17 (SBS17) in EAC and gastric cancers (31, 32). SBS17 is subdivided into SBS17a, characterized by T > C conversion in the CTT trinucleotide context, and SBS17b, defined by T > G substitution in any of the NTT trinucleotide contexts (32). Cancer patients treated with 5-fluorouracil acquire SBS17-specific mutations (33), and it was previously proposed that oxidized deoxyguanosine triphosphate (dGTP) nucleotides contribute to their generation (34). The causative insult leading to the acquisition of SBS17 in BE, EAC, or gastric cancers may be related to gastric-esophageal reflux.

Here, we use a variety of molecular and BE organoid-based experiment in a search for biomarkers for the different BE stages.

## Results

### Molecular Characterization of BE Organoids.

Glandular structures of the BE epithelium were isolated from native biopsies, as previously described for gastric glands (35), and organoid cultures were established using small intestinal culture medium after some minor adaptations (*SI Appendix*, Fig. S1 and *Methods*) (36). BE organoids displayed a cystic morphology with an inner lumen similar to gastric and small intestinal cultures, whereas dense structures, which were previously reported for normal squamous esophageal epithelium (37), were not observed (*SI Appendix*, Fig. S1*B*). BE cells supported the growth of organoids from single-cell suspensions, which allowed clonal expansion of single-cell clones. Such single-cell-derived cultures are ideally suited to investigate the mutational landscape by WGS as previously reported for colon, small intestine, and liver organoids (38) and colorectal tumor cultures (39).

**Fig. 1.**
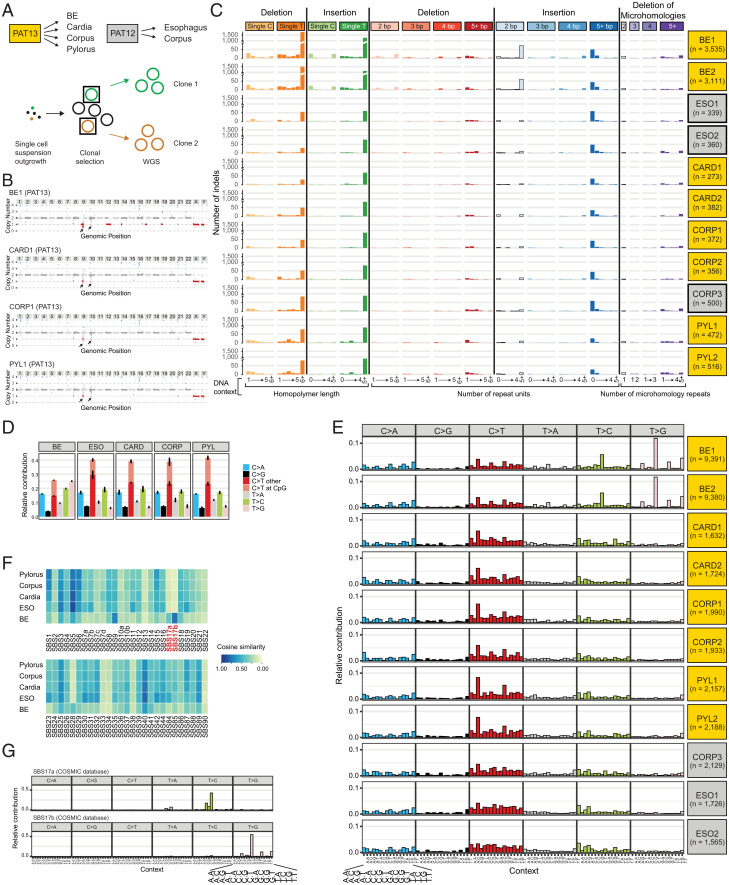
(*A*) Overview of biopsy collection for PAT12 and PAT13 and schematic representation of the clonal organoid expansion for WGS. Data derived from PAT12 or PAT13 are indicated by gray or yellow shading, respectively (boxes in *A*, *C*, and *E*). (*B*) Examples of the karyotyping of the respective clone 1 derived from the BE, gastric cardia, corpus, or pylorus region. Black arrows indicate regions with low sequence coverage in all samples and controls. Due to the unreliable detection of such repetitive sequence reads, these regions were not considered for the overall karyotype determination (see also *SI Appendix*, Fig. S2). (*C*) INDEL patterns for the sequenced organoid clones. n refers to the number of detected INDELs in each clone. (*D*) Summary of single-nucleotide conversions for the sequenced organoid clones. (*E*) Overview of trinucleotide signatures for the sequenced organoid clones. n refers to the number of detected SBS in each clone. (*F*) Cosine similarities of single-nucleotide conversion to the COSMIC SBS signatures. Please note that SBS9 and SBS17b share similar mutational features, which leads to the relative enrichment of SBS9 in cells with SBS17b patterns in the cosine similarity plot. (*G*) Trinucleotide signatures of SBS17a (*Top*) and SBS17b (*Bottom*), as defined by the COSMIC database.

We clonally expanded epithelial cells from fresh biopsies obtained from different anatomic regions of two patients. From patient 13 (PAT13) we collected one BE biopsy, diagnosed as LGD, and three healthy gastric biopsies from the gastric cardia, corpus, and pylorus regions (Fig. 1*A*). From PAT12 we established organoids from the esophageal squamous epithelium and gastric corpus region (Fig. 1*A*). WGS data analysis revealed stable diploid karyotypes for all nondiseased clones, whereas partial losses on chromosomes 9, 12, 14, 17, and 19 were found in the BE clones (Fig. 1*B*, *SI Appendix*, Fig. S2, and Dataset S1). The appearance of CIN in the BE clones is in agreement with existing literature that BE cells from LGD tissue accumulate chromosome alterations (25–27). Additional in-depth analyses focusing on insertions and deletions (INDELs) identified on average 400 events per clone derived from nondiseased epithelia (Dataset S2). The highest numbers within PAT13 were detected in the clones of the gastric pylorus, followed by the corpus and the cardia region (>500, ∼350, and ∼300 INDELs, respectively), and within PAT12 the nondiseased esophageal clones carried significantly lower numbers of INDELs compared to cells derived from the gastric corpus region (∼350 versus 500 INDELs) (Fig. 1*C* and Dataset S2). Almost 10 times more INDELs were identified in the two BE clones (3,525 and 3,111). The largest increase in absolute numbers was recorded for single T INDELs, in particular in poly-T stretches (nondiseased gastric and esophageal clones: 50 to 150; BE clones: 1,000 to 1,700) (Fig. 1*C* and Dataset S2). These INDEL patterns correspond to the COSMIC INDEL signatures ID1 and ID2 (*SI Appendix*, Fig. S3*A*) (32). BE clones also revealed higher incidences of double-nucleotide insertions after repeat stretches (Fig. 1*C*), and the most frequent dinucleotide insertions were AT and TA sequences (Dataset S2).

Next, we concentrated our analysis on single-base substitutions (SBS). For the clones derived from nondiseased epithelia, the nucleotide conversion patterns were very similar, with the highest incidence rates being observed for C > T conversions (Fig. 1*D*). The number of detected SBS per anatomic regions followed a similar trend as for the incidences of INDELs. The highest numbers were observed in the gastric pylorus, followed by the corpus and cardia region in PAT13 (∼2,150, ∼1,950, and ∼1,700, respectively), and the nondiseased esophageal cells of PAT12 carried fewer SBS than the corresponding gastric corpus cells (∼1,650 and ∼2,100) (Dataset S3). Significantly more SBS were detected in the BE clones compared to the nondiseased control regions (∼9,380 per clone), and the most obvious difference was observed in the frequency of T > G conversions (Fig. 1*D*, *SI Appendix*, Fig. S2*C*, and Dataset S3). These alterations correlated with the appearance of the two subsignatures of SBS17, SBS17a and SBS17b (Fig. 1 *E*–*G* and *SI Appendix*, Fig. S3*B*). Moreover, there was a higher likelihood of an A or T two nucleotides upstream of any T > C or T > G conversion (*SI Appendix*, Fig. S3*C*). While the SBS17 signatures were previously associated with BE (24, 25), we extended our analysis to the adjacent nondiseased control tissue. As gastroesophageal reflux is a key factor for the development of BE, it is believed to be also associated with the acquisition of SBS17 mutations (31). Moreover, these patterns are also detected in gastric cancers (32). We therefore analyzed the COSMIC signatures in the nondiseased gastric and esophageal tissues and yet could not detect any evidence of SBS17-specific mutations in clones derived from nondiseased control biopsies (Fig. 1 *E* and *F*). This excluded the possibility that such mutations could arise in nondiseased control cells located in close proximity to BE. Hence, BE cells appear to be more sensitive to mutational processes leading to SBS17-specific alterations. As a hallmark of BE development is the transformation to a columnar epithelium, we were wondering whether these morphological changes coincided with the acquisition of SBS17-characteristic mutations. We hypothesize that these mutations could occur at a later stage during BE progression, which we further investigated at the single-cell level in fresh biopsies.

### DNA Alterations within the BE Epithelium.

For this purpose, we applied the recently developed single-cell DNA sequencing (scDNAseq) technology (40). This method is ideally suited to identify CINs in single cells and SBS in cell clusters. The acquisition of CIN is an important hallmark of the progression toward EAC (28, 29), and previous studies correlated the degree of CIN with advanced dysplastic stages based on loss-of-heterozygosity analyses (26, 27).

**Fig. 2.**
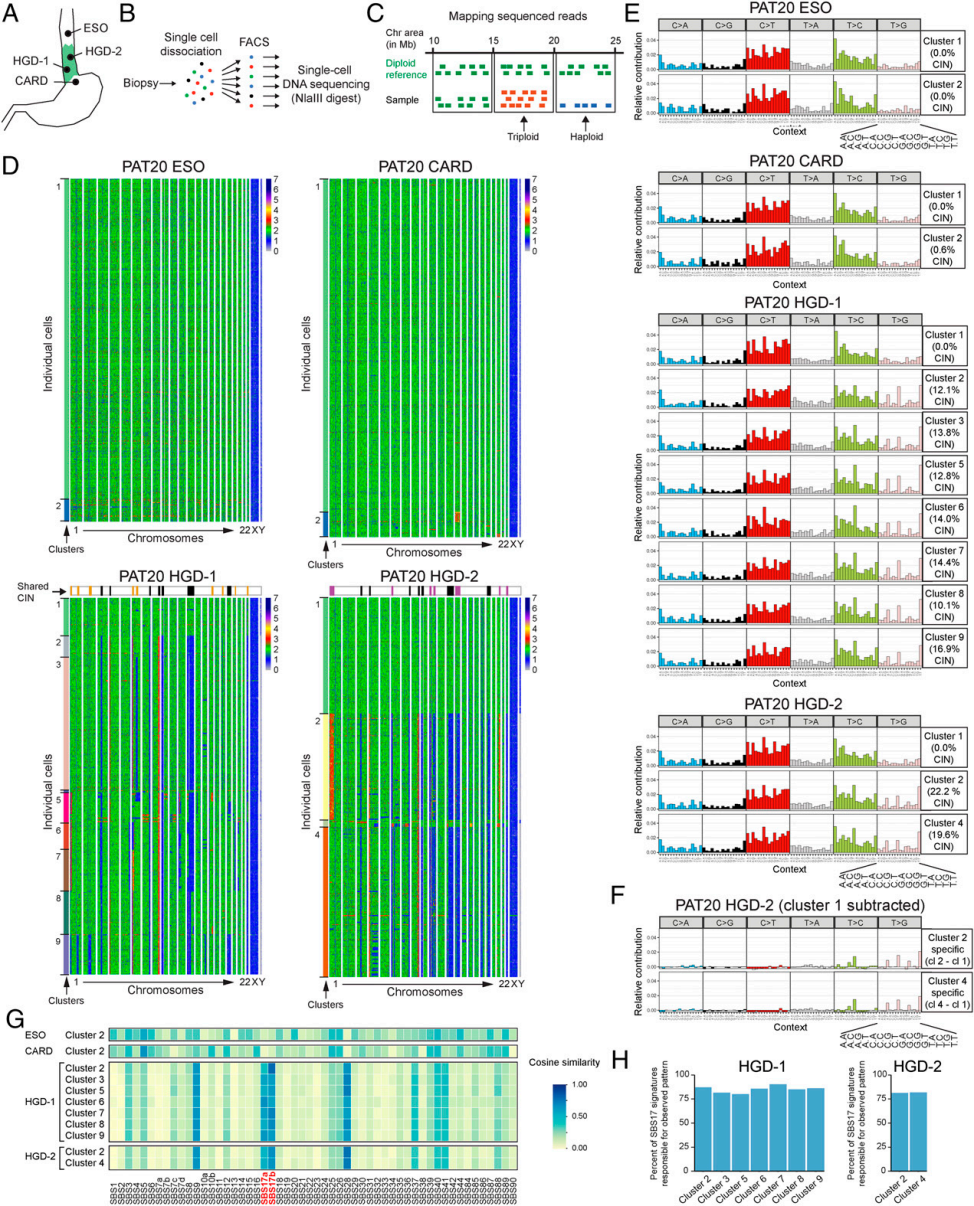
(*A* and *B*) Overview of the locations for biopsy collection (*A*) and schematic representation of single cell generation and isolation by FACS sorting prior to scDNAseq (*B*). (*C*) Schematic representation of scDNAseq data analysis to identify chromosomal unstable regions. (*D*) Heat maps showing chromosomal stability at a single-cell level for the indicated biopsies. The *x* axis indicates individual chromosomes. The *y* axis contains individual cells, which were clustered bioinformatically as indicated by the first column on the left (see Dataset S5). Black boxes on top of the HGD-1 and HGD-2 heat maps mark shared CIN regions between the two BE biopsies, whereas yellow and purple boxes highlight biopsy-specific alterations. (*E*) Overview of trinucleotide signatures observed for the identified cell clusters in individual biopsies as shown in *C*. (*F*) Trinucleotide pattern for CIN cell cluster of PAT20 HGD-2 biopsy after subtracting the trinucleotide pattern of the respective CS cell cluster. (*G*) Cosine similarity plot showing the resemblance to COSMIC SBS for each cluster after subtracting the trinucleotide pattern of the biopsy-internal CS cell cluster of PAT20. The signatures SBS17a and SBS17b are highlighted in red. Please note that SBS9, SBS17b, and SBS28 share similar mutational features, which leads to the relative enrichment of SBS9 and SBS28 in cells with SBS17b patterns in the cosine similarity plot, particularly in cells with low sequencing coverage. (*H*) Signature reconstitution plots for SBS17a and SBS17b. These plot show to what degree SBS17a and SBS17b can explain the observed trinucleotide pattern in the CIN clusters.

**Fig. 3.**
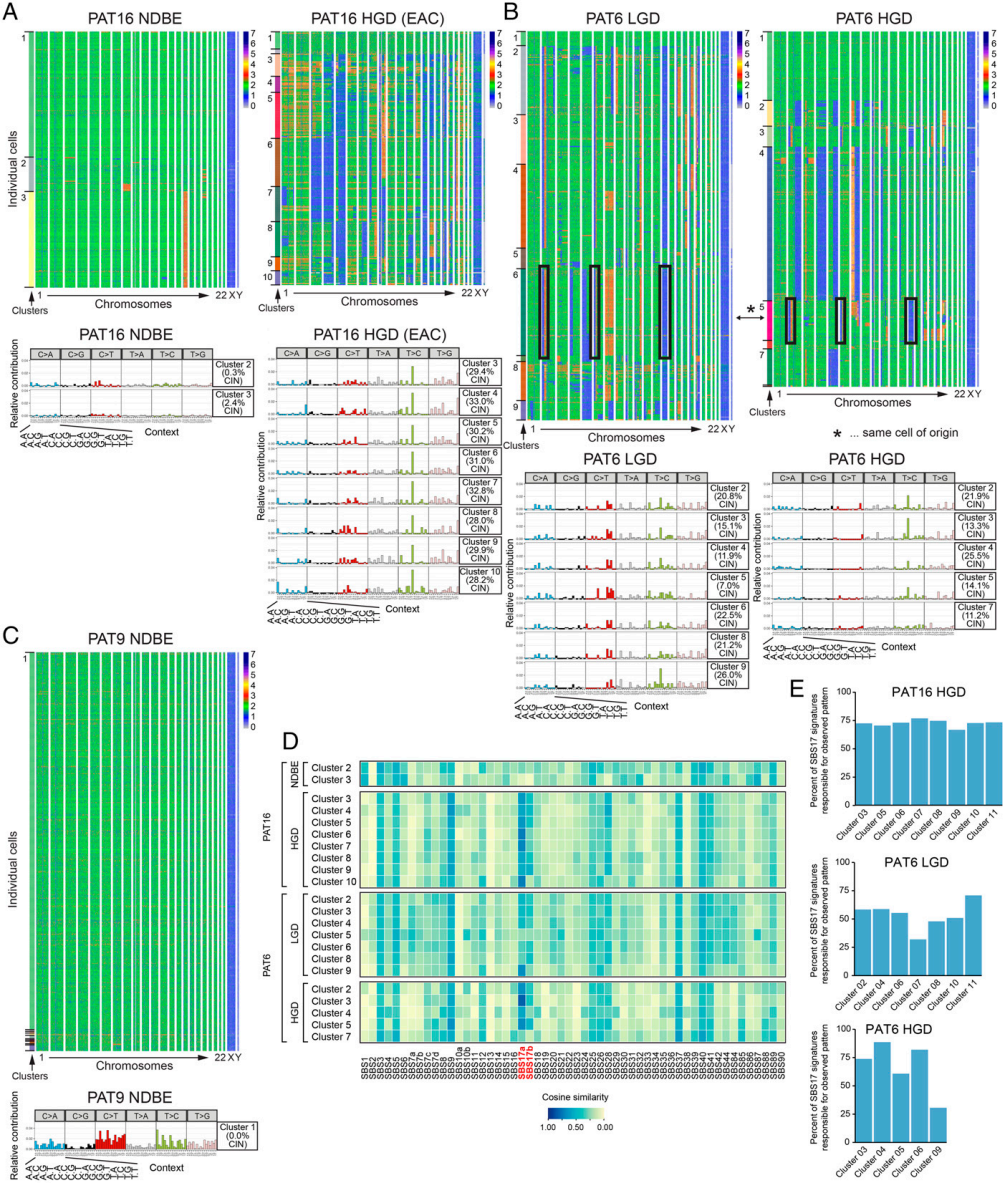
(*A*–*C*) Heat maps showing chromosomal stability at the single-cell level. The *x* axis indicates individual chromosomes and the *y* axis individual cells. Cell clusters are shown on the left (*Top*). The cells from LGD and HGD BE biopsies of PAT6 (*B*) shared very few chromosomal alterations (black boxes and asterisk), indicating that the majority of cells are derived from at least two different ancestral cells. Overview of trinucleotide pattern for the identified CIN cell clusters after subtracting the trinucleotide pattern calculated for the respective CS cell cluster (*Bottom*). The signatures SBS17a and SBS17b are highlighted in red. (*D*) Cosine similarity plot showing the resemblance to the COSMIC SBS for each cluster after subtracting the trinucleotide pattern of the biopsy-internal CS cell cluster (PAT6 and PAT16). (*E*) Signature reconstitution plots for SBS17a and SBS17b. These plot show to what degree SBS17a and SBS17b can explain the observed trinucleotide pattern in the CIN clusters.

We performed scDNAseq experiments with fresh biopsies from patients with different BE stages (Dataset S4, Fig. 2 *A* and *B*, and *Methods*). Histological analysis of adjacent biopsies, which were graded by expert pathologists, was used as an approximation to determine the BE stages of the biopsies analyzed. From one patient (PAT20) we collected biopsies from four different anatomical regions, including the nondiseased esophageal and gastric cardia epithelium as well as two high-grade BE biopsies (HGD-1 and HGD-2), which were macroscopically separated by at least 5 cm from each other (Fig. 2*A*). Cells from the nondiseased esophageal and gastric tissue were largely chromosomally stable (CS), whereas both BE biopsies carried a multitude of chromosomal gains and losses (Fig. 2 *B*–*D*). Some alterations were shared between HGD-1 and HGD-2 (Fig. 2*D*, black boxes on top of heatmaps), which point to a common ancestral clone. Additionally, each biopsy had acquired unique alterations (Fig. 2*D*, purple and yellow boxes on top of the heatmaps), which indicated further subclonal evolution. Cells were bioinformatically clustered based on their genome stability (Fig. 2 *D*, *Left*, Dataset S5, and *Methods*). The nondiseased gastric and esophageal biopsies yielded two clusters of CS cells, whereas more cell clusters were identified in the BE biopsies. Among the eight (or three larger) cell clusters for HGD-1 and HGD-2, one was always CS. The SBS patterns were calculated for all identified clusters and revealed comparable SBS patterns for the CS cells and the nondiseased control epithelium (Figs. 2*E* and 1*E*). These CS cells are unlikely contaminations by nondiseased epithelial or mesenchymal cells, since the applied cell isolation strategy (*SI Appendix*, Fig. S1*A*) did not yield such cells (see also scRNAseq experiments below and *Methods*). We did, however, not exclude immune cells in the fluorescent activated cell sorting (FACS) sorting step. As we performed scRNAseq with sorted cells of replica plates, we could estimate the potential immune cell contamination, which was generally <6% of the analyzed cells, while the identified CS cluster was significantly larger than these percentages (see Dataset S6). The trinucleotide pattern of all analyzed CIN cell clusters revealed signs of SBS17, which was further highlighted by subtracting the trinucleotide pattern of the CS cell cluster (Fig. 2 *E* and *F* and *SI Appendix*, Fig. S3*A*). The resulting cluster-specific patterns also correlated with the available COSMIC SBS signatures, which confirmed a good match with SBS17a and SBS17b (Fig. 2*G*). When focusing only on SBS17a and SBS17b, these signatures explained >75% of the observed mutational pattern (Fig. 2*H*). Most strikingly, we observed a correlation between CIN and the appearance of SBS17. Since we did not have matching germline controls for identifying SNPs, we removed all germline variants, described in the public SNP database, and SNPs that we could identify in all our scDNAseq samples (see *Methods*). To exclude that SBS17-specific SNPs were removed in the latter filtering step, we calculated their trinucleotide pattern, where SBS17-characteristic mutation patterns were absent (*SI Appendix*, Fig. S4*B*). To investigate the relationship between CIN and SBS17 in more depth, we focused our analysis on biopsies from other patients. All NDBE biopsies (*n* = 4) were CS, and none revealed traces of the SBS17 signatures (Fig. 3 and *SI Appendix*, Fig. S4 *C*–*E*). Among the LGD biopsies (*n* = 3), one patient (PAT6) carried excessive CIN, whereas the other two patients (PAT15 and PAT19) did not, and the SBS17 mutations were detected only in the CIN cells of the LGD biopsy of PAT6 (Fig. 3*B* and *SI Appendix*, Figs. S4 *D* and S5 *C* and *D*). We also obtained biopsies from three patients with HGD (PAT6, 14, and 20; Figs. 2*D* and 3*B* and *SI Appendix*, Fig. S5*A*) and one patient (PAT 16), who additionally also developed focal EAC (Fig. 3*A*). The majority of the cells within these biopsies were CIN and, in three of the four patients, the SBS17a and SBS17b signatures were detected (Fig. 3 *A* and *B* and *SI Appendix*, Fig. S5*A*). Biopsies of PAT20 revealed clear alterations characteristic for both signatures, while SBS17a was more pronounced in cells analyzed from PAT6 and PAT16 (Figs. 2 *E*–*G* and 3 *A*, *B*, and *D* and *SI Appendix*, Fig. S4*A*). Only the HGD biopsy of PAT14 did not show any clear evidence of SBS17 (*SI Appendix*, Fig. S5*A*). Signature reconstitution plots revealed that the SBS17a and SBS17b could explain most of the observed mutational patterns in PAT6, PAT16, and PAT20 (Fig. 3*E*). Of note, the identification of SNPs in scDNAseq data are difficult due to technical limitation (see *Methods*), which explains some of the noisy trinucleotide patterns before and after the subtraction of the respective CS cluster (Figs. 2 and 3 and *SI Appendix*, Fig. S5). Moreover, subtraction of the mutation pattern of individual CS cell cluster, which also showed variability, could lead to artificial patterns, as indicated by the C > T conversions (Fig. 3*B*, *SI Appendix*, Fig. S4*D*, and *Methods*). To normalize for such fluctuations in CS cells, we calculated a common CS trinucleotide pattern based on all analyzed cells, which was subtracted from every cell cluster in all patients, and used the remaining pattern for cosine similarity calculation. These analyses confirmed the absence of SBS17 in CS cells (*SI Appendix*, Fig. S4*F*).

The striking correlation between CIN and the presence of the SBS17-specific mutation patterns is best illustrated by biopsies obtained from the same patient (PAT16), where only CIN cells within the HGD biopsy had acquired T > C and T > G conversion, whereas cells in the matching NDBE biopsy did not (Fig. 3*A*). Hence, our high single-cell-resolution analysis of BE biopsies identified a strong correlation between CIN and SBS17 in BE, in contrast to previous reports based on WGS of entire biopsies (24, 25).

### Gene Expression within the BE Epithelium.

**Fig. 4.**
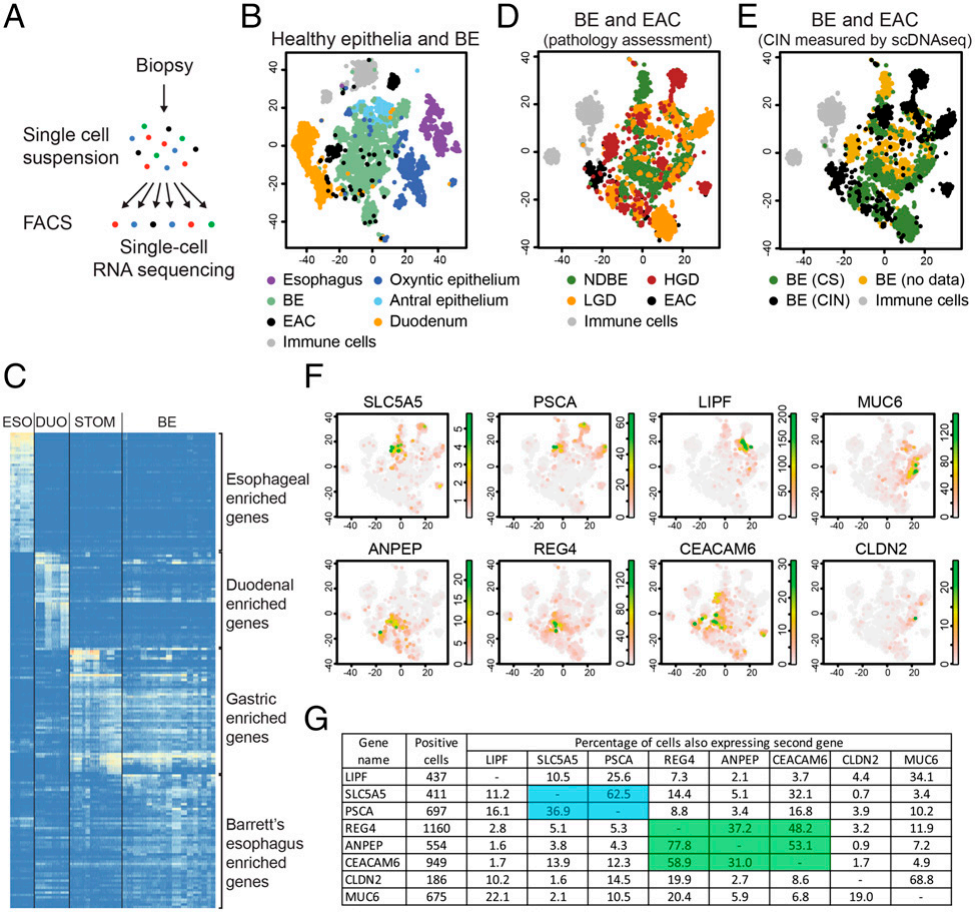
(*A*) Overview of scRNAseq protocol. (*B*) t-SNE map displaying the scRNAseq data of all nondiseased control samples and all analyzed BE biopsies. (*C*) Heat map displaying the expression of the most differentially expressed genes, clustered according to their expression in the esophagus (ESO), small intestine (DUO), stomach (STOM), and BE. (*D* and *E*) t-SNE maps displaying the scRNAseq data of all the BE and EAC samples. Cells were colored based on the pathology assessment of the respective biopsies (*D*) or the CIN or CS state of the biopsies, as determined by scDNAseq experiments (*E*). (*F*) t-SNE maps showing the expression of *SLC5A5*, *PSCA*, *LIPF*, *MUC6*, *ANPEP*, *REG4*, *CEACAM6*, and *CLDN2*. The reference t-SNE plot is shown in *D*. (*G*) Table showing the overlap in expression of individual genes within the same cell in the scRNAseq data. Left two columns indicate the analyzed genes and the number of cells, which were positive for the indicated genes. Columns 3 to 10 show the percentage of cells that coexpress the candidate gene (column name) and gene of interest (row name). The percentages refer to cells with positive expression of the gene of interest (in column 1).

While dysplastic BE stages are correlated with CIN (25–27), their histological distinction from NDBE remains challenging, and the associated gene expression changes are still largely unknown. Therefore, we performed scRNAseq experiments with biopsies from 14 BE patients, which include 7 NDBE, 3 LGD, and 5 HGD stages as well as 2 EACs according to the assessment by expert pathologists (Fig. 4*A* and Datasets S4 and S7). In our initial analysis, we also included our previously published reference dataset of nondiseased esophageal, gastric, and duodenal tissue as nondiseased control samples (Fig. 4*B* and Datasets S4 and S7) (37). The nondiseased controls formed separate clusters in the t-SNE map and showed distinct gene expression profiles (Fig. 4 *B* and *C*, *SI Appendix*, Fig. S6*C*, and Dataset S8) (37). The gene expression pattern of BE cells was unique but showed the best transcriptional overlap with the gastric epithelium, while sharing some gene expression similarities with the small intestine (Fig. 4*C*, *SI Appendix*, Fig. S6*C*, and Dataset S8). These data are in contrast to a previous study which observed no significant transcriptional overlap between BE and gastric cells (17). This study furthermore reported *LEFTY1*, *OLFM4*, *SPINK4*, *ITLN1*, *TFF3*, and *KRT7* as BE-specific genes (17). In our dataset, we also observed enriched expression of these genes in BE cells (*SI Appendix*, Fig. S6*A*) and thus concluded that our data should be quite similar to these published data (17). The fact that we separately analyzed the oxyntic and antral epithelia of the stomach may explain the divergent conclusions as the antral epithelium, which was previously not analyzed (17), showed the best transcriptional overlap with BE cells (Fig. 4*B*).

We next focused our analysis on BE and EAC biopsies and color-coded the cells based on their pathology assessment on the respective t-SNE map (17) (Fig. 4*D*). The gene expression profiles were quite diverse and some of the cell clusters were even derived from individual biopsies and did not coincide with cells from other patients (Fig. 4*D* and *SI Appendix*, Fig. S6*D*). Batch effects could be largely ruled out as the majority of samples were processed simultaneously, and the immune cells from all sequenced samples overlapped in a single cell cluster (*SI Appendix*, Fig. S6*D*). Alternative explanations for suboptimal clustering could be 1) clustering artifact due to CIN and 2) the inherent problem of pathology staging.

CIN may lead to low-level dose-dependent gene expression changes that could influence cell clustering. For some biopsies, we simultaneously performed scRNAseq and scDNAseq experiments by analyzing replica plates generated by the same FACS-sorting experiment (Dataset S4). Based on the scDNAseq data, we marked CIN and CS cells from the different biopsies in black or green, respectively (Fig. 4*E*). This representation of the data revealed many biopsy-specific CIN cell clusters. To prevent a CIN-induced bias of clustering, we restricted our analysis only to genes located in genomic stable regions, present in all biopsies. To validate such an approach, we focused on the scRNAseq dataset of the PAT20 biopsies (*SI Appendix*, Fig. S6*E*). The separate clustering of nondiseased control tissue was unaffected by the removal of genes located in the CIN regions, whereas cells from HGD-1 and HGD-2 biopsies merged into one large cluster (*SI Appendix*, Fig. S6*E*). This is in agreement with our previous observation that these cells may be derived from the same ancestral cell clone (Fig. 2*D*). However, such an approach was not applicable for a combined analysis of all sequenced biopsies, since the CIN regions were quite heterogenous between them, and hardly any gene would be left for analysis if these areas were excluded. We therefore focused our analysis on all NDBE biopsies and the dysplastic biopsies of PAT6 and PAT20 (*SI Appendix*, Fig. S6 *F* and *G*). The previously observed biopsy-specific clustering improved and yielded a rather uniform CIN cell cluster (*SI Appendix*, Fig. S6 *F*–*H*), which revealed an enrichment for genes associated with CIN cells (*SI Appendix*, Fig. S6*I*). The gene expression pattern of three of these genes, *MUC1*, *CLDN18*, and *KLF2*, was validated by RNA in situ hybridization experiments on endoscopic resection specimen (*SI Appendix*, Fig. S7*B*). They were selectively expressed in the BE epithelium but were not restricted to individual BE stages. For instance, *MUC1* showed some specificity for dysplastic stages in patient HIS-PAT7 but was detected at all BE stages in HIS-PAT1 to HIS-PAT4. These genes were selected based on their location in CS regions of the genome. It is, however, possible that their expression is indirectly influenced by the deregulation of genes located in CIN regions. This could explain the discrepancy in gene expression measured by RNA in situ hybridization on histological sections and scRNAseq data analysis of biopsies.

Another explanation for the inconsistent BE stage-specific clustering could be the somewhat “imperfect” pathology staging procedure. Dysplasia in BE biopsies is often focal and the majority of the adjacent epithelium in the same biopsy is frequently composed of NDBE or other BE stages (5, 6, 41). Biopsies are staged based on the most advanced stages even if these cells represent only a minority within the biopsy. In scRNAseq, we analyzed, however, the entire biopsy, including all the distinct BE stages. This could explain the frequent mixing of cells derived from different BE stages in our cluster calculation.

**Fig. 5.**
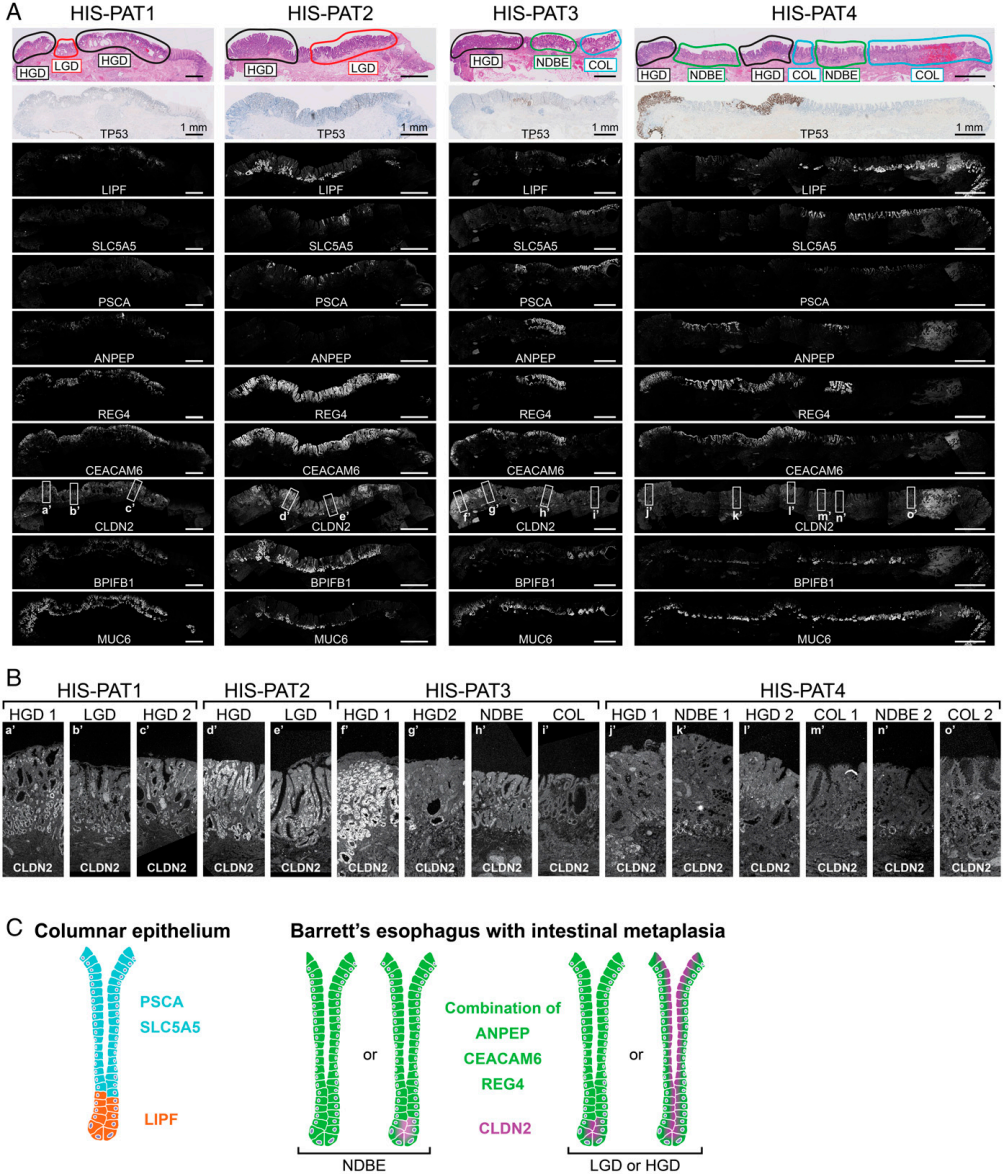
(*A*) Four histological resection specimen containing different regions of BE were analyzed for the expression of *LIPF*, *SLC5A5*, *PSCA*, *ANPEP*, *REG4*, *CEACAM6*, *CLDN2*, *BPIFB1*, and *MUC6* by RNA in situ hybridization. The first row shows the hematoxylin/eosin staining indicating the pathology assessment of the BE stages, and the second row displays the TP53 antibody staining obtained from the pathology department. (Scale bars: 1 mm.) (*B*) *CLDN2* expression is shown at higher magnification for individual BE stages as indicated by rectangles in *A*. (*C*) Proposed expression patterns of the different genes in the columnar epithelium and BE with IM, including a potential distinction between NDBE and dysplastic (LGD and HGD) stages.

All these points argue for an unbiased analysis independent of the initial pathology staging outcome. Therefore, we selected 17 marker genes with different expression patterns in individual cell clusters (*SI Appendix*, Fig. S7*A*) and validated their expression by RNA in situ hybridization experiments on endoscopic BE resection specimens. While all genes yielded a positive signal in at least one histological section, only seven genes enabled a reliable distinction between different stages (Fig. 5 and *SI Appendix*, Fig. S8). Within the COL epithelium without IM, *PSCA* and *SLC5A5* were expressed in the surface epithelium, while *LIPF* was simultaneously expressed in the deeper gland tissue (Fig. 5*A* and *SI Appendix*, Fig. S8*A*). Coexpression of *ANPEP*, *CEACAM6*, and *REG4* marked regions of BE with IM. A similar trend was also observed in our scRNAseq data, where *REG4* was more likely to be expressed in ANPEP- or CEACAM6-positive cells in contrast to LIPF-, PSCA-, or SLC5A5-positive cells (Fig. 4*G*). The expression of these two gene sets was largely mutually exclusive, while PSCA was least specific in our histological staining, since it was also occasionally expressed in BE with IM. *REG4* and *CEACAM6* are particularly enriched in areas containing IM but cannot be used to distinguish between NDBE and dysplastic stages. Only the expression of *CLDN2* revealed some specificity for dysplastic stages (Fig. 5 *A* and *B* and *SI Appendix*, Fig. S8 *A* and *B*). *CLDN2* expression was hardly detectable in COL and was very weak, if expressed at all, in deeper glands of NDBE. Its expression often increased in LGD and HGD stages and spread throughout the entire glands (Fig. 5*B* and *SI Appendix*, Fig. S8*B*; HGD area of HIS-PAT1-3 and HGD/LGD of HIS-PAT5-7).

Based on the validation on these “training” slides, we propose the following model (Fig. 5*C*). An area expressing *SLC5A5* and *PSCA* in the surface epithelium and *LIPF* in lower glands corresponds to COL, while a region positive for *ANPEP*, *CEACAM6*, and *REG4* refers to BE with IM, consistent with largely mutually exclusive expression of these two gene set at these BE stages. Additionally, an increase and spreading of CLDN2 expression highlight potential dysplastic areas. It is important to note that, based on the expression data of the provided training slide (Fig. 5 and *SI Appendix*, Fig. S8), an absence of CLDN2 staining cannot be interpreted as an unambiguous sign for the absence of dysplasia.

Next, we tested the validity of these markers on six BE biopsies, which were previously diagnosed as NDBE, “indefinite for dysplasia,” or LGD (*SI Appendix*, Fig. S9). All analyzed histological sections, except for one (HIS-PAT8), expressed the IM markers. The histological section of HIS-PAT8 was, however, downgraded to COL after closer inspection of the stained biopsy (*SI Appendix*, Fig. S9). COL and BE with IM were detected side by side in the histological section of HIS-PAT12, which was also confirmed after reanalysis of the provided hematoxylin/eosin staining at higher magnification (*SI Appendix*, Fig. S9*B*). There was also a good correlation of *CLDN2* staining with one NDBE and both LGD biopsies (*SI Appendix*, Fig. S9 *A* and *B*). Overall, we observed a good correlation between the staining patterns of our newly identified marker genes and the associated pathology assessment.

## Discussion

We have used different sequencing techniques to characterize the BE epithelium at the single-cell level and to identify molecular features characteristic of the individual BE stages. By performing WGS of single-cell-derived organoid cultures we observed selective acquisition of T > G and T > C conversion in BE clones that correspond to the previously described SBS17a and SBS17b (24, 25, 31, 32). While some mutational signatures such as SBS1 and SBS5 accumulate as a result of aging, SBS17-related alterations were not detected in esophageal cells of individuals (>85 y of age) (42, 43). In agreement with these observations, SBS17-specific alterations were absent in matching nondiseased esophageal and gastric tissues including the adjacent cardia region, located on the gastric side of the gastroesophageal junction. They were, however, specifically detected in the BE epithelium. Either the gastric epithelium is better protected against the causative mutagenic process by the local mucosal layer or the responsible insult is not present in the stomach. To gain more insight into the nature of SBS17-related mutations, we performed scDNAseq of epithelial biopsies to investigate DNA alterations at the single-cell level. While the overall genome coverage of this approach is still relatively low and does not allow the reliable identification of individual mutations in these cells, it enabled the detection of T > C and T > G conversions in cell clusters. Interestingly, not all cells within the BE biopsy acquired these mutations and were thus not equally affected. The SBS17 signatures were never detected in CS cells but were consistently observed in CIN cells. Our findings are in contrast with previous studies reporting that all BE cells including the CS cells acquire SBS17 (24, 25). In these studies, BE and EAC biopsies were collected from the same patients, who already developed EAC. The entire endoscopic biopsy was sequenced, including the associated mesenchymal and immune cells. Such cellular contaminations could interfere with the assignment of the CIN status, which may explain the discrepancy between these published and our data. A recent study analyzed BE biopsies by WGS and grouped them based on their ability to develop EAC in follow-up examinations (44). Two patients did not progress past the stage of the initial BE diagnosis, and the SBS17 signatures were absent in the initial biopsies and the follow-up biopsies after 3 or 4 y, respectively (44). These findings agree with our observations that not all BE biopsies acquire SBS17-specific mutations, in particular those with a low probability of tumor progression. In contrast to the sequencing of the entire biopsy of previous studies (24, 25, 44), we have now provided a high single-cell-resolution analysis of BE biopsies, which identified a strong correlation between the emergence of CIN and SBS17. The likelihood of acquiring CIN is known to increase at advanced dysplastic BE stages (26, 27), which is accompanied by the appearance of SBS17-specific mutations, as shown here. Although no conclusions about a causal relationship between CIN and SBS17 can be drawn, future work will be required to address this question.

Recent publications shed some light on the etiology of the SBS17a and SBS17b signatures. Incorporation of oxidized dGTP into the DNA favors the SBS17-specific T > G conversions (34), and such mutations were also observed in patients treated with 5-fluorouracil (33). The causative insult leading to these damages in patients is, however, still unknown. In some patients we observed a prevalence for SBS17a, while SBS17a and SBS17b were simultaneously found in other patients. These findings may point to different mechanisms generating these two signatures. How gastric reflux is involved in these processes remains unclear. Most BE patients are treated with proton pump inhibitors, which abolish the damaging effects of the harsh acidic environment. Such patients are nonetheless capable to progress and develop EAC. Alternative explanations for the induction of such mutagenic processes in BE could be environmental changes in metabolite or microbiome composition or loss of cell-intrinsic protective functions, as exemplified by gene mutations of TP53, functioning as a gatekeeper of genome integrity.

We systematically characterized single-cell gene expression profiles across different BE stages. Bioinformatic analysis of our datasets identified two gene sets that allowed the distinction between COL and BE with IM. Coexpression of *SLC5A5* and *PSCA* in the surface epithelium and simultaneous expression of *LIPF* in the deeper gland region was a good indicator for the presence of columnar epithelium (“COL markers”). These staining patterns mimic gastric glands as *SLC5A5* is expressed in gastric foveolar cells, *LIPF* in chief cells, and *PSCA* at low levels in all gastric cell types (37). However, other genes characteristic of these gastric cell types were not coexpressed in cells of the BE epithelium, which points to a specific up-regulation of these selected genes. Of note, the gastric-specific gene *MUC6* was detected in all BE resection specimens analyzed (Fig. 5*A* and *SI Appendix*, Fig. S8*A*). *MUC6* and *LIPF* are known to be coexpressed in cells of the gastric antral epithelium (37), and such a pattern was occasionally observed in our BE samples. Additionally, the expression of *MUC6* is also up-regulated in spasmolytic polypeptide-expressing metaplastic regions (SPEM) in gastritis patients (45). We also observed MUC6 expression in deep glandular structures of IM-containing BE epithelium, indicating some similarities with SPEM. The expression of “COL markers” was mutually exclusive to that of the second gene set consisting of *ANPEP*, *CEACAM6*, and *REG4*, which were predominantly expressed in BE areas containing IM (“IM markers”), including NDBE and dysplastic stages. ANPEP and CEACAM6 were previously reported to be either up- or down-regulated in dysplastic stages, respectively (46, 47). Although we also observed specific expression of these genes in BE with IM, our data could not confirm a trend for either dysplastic or nondysplastic stages. The expression of *REG4* was most reliable in identifying BE areas with IM. While *REG4* was previously identified by microarray analysis to be expressed in BE without distinguishing between NDBE and dysplastic stages, its expression level was not yet validated by histological analysis or correlated to different BE stages (48). *REG4* was often found to be coexpressed together with *CEACAM6* and/or *ANPEP* (together “IM markers”) in BE regions with IM. Based on histological staining, *PSCA* was the only COL marker that showed some ambiguity and was occasionally coexpressed with IM markers. However, the simultaneous expression of *SLC5A5*, *PSCA*, and *LIPF* as described for COL epithelium never overlapped with IM markers. While the COL and IM markers were good predictors for COL and BE with IM, they did not distinguish between dysplastic and nondysplastic stages.

*CLDN2* was the only gene with a more selective expression pattern in dysplastic stages. CLDN2 is a component of tight junctions and forms gated paracellular channels to allow small positively charged ions to cross between cells (49). We observed hardly any expression of *CLDN2* in COL and NDBE, which is also in agreement with a previous publication (50). If present at all, its expression was weak and localized to deeper gland areas. In dysplastic stages, the expression of *CLDN2* increased and could also be found throughout the entire epithelium. *CLDN2* expression was previously reported to be up-regulated in EAC (51). However, the literature about CLDN2 expression in BE is controversial, as studies using different antibodies reported the presence (52) or absence (51) of *CLDN2* expression in BE. While analyzing *REG4* expression, we observed a discrepancy between antibody staining and RNA in situ hybridization, as RNA in situ hybridization proved to be more sensitive and reliable in detecting *REG4* expression, which furthermore correlated more consistently with the pathology assessment. The use of RNA in situ hybridization for our histological confirmation experiments may also explain why *CLDN2* expression correlated better with the dysplastic stages compared to previous antibody staining analyses. *CLDN2* expression may be a useful addition to the currently used TP53 staining for identifying dysplastic BE stages. Our study validated *CLDN2* expression only on a limited number of histological sections, which does not yet allow the conclusion that the absence of *CLDN2* staining can be used as an unambiguous exclusion criteria for dysplastic BE. In the future, *CLDN2* and the BE-specific gene sets identified in this study will need to be validated in larger patient cohorts to confirm their specificity for the different BE stages and their usefulness for pathological assessment.

### Human Patients.

The study was approved by the ethical committee of the University Medical Center Utrecht (UMCU) and was in accordance with the Declaration of Helsinki. It is also according to Dutch law and compliant with all relevant ethical regulations regarding research involving human participants. A standard biopsy forceps was used to take biopsies of BE, healthy esophageal squamous or healthy gastric cardia epithelial tissue. Biopsies from BE patients were only sampled if the BE segment (>3 cm) was clearly distinguishable from the surrounding healthy squamous epithelium during endoscopic examination and clearly located above the gastroesophageal junction. Special care was taken to only sample BE epithelium, minimizing the risk of cellular contamination from surrounding nondiseased epithelium. Gastric cardia biopsies were taken within the 1-cm rim below the gastroesophageal junction, identified based on the first gastric folds and the palisade vessels. All included individuals signed an informed consent and their personal information was anonymized. A detailed overview of sex, age, and diagnosis is provided in Dataset S1.

Sections of formalin-fixed, paraffin-embedded human endoscopic resection specimen or biopsies was obtained from the pathology archives at the UMCU and they were anonymized according to the guidelines of the UMCU’s Research Ethics Committee (53).

## Methods

See Dataset S9 for resources and reagents.

### Processing of Human Biopsies.

BE and gastric cells from human biopsies were isolated as previously described (35, 37). The columnar epithelium or gastric glands were squeezed out of the biopsies, which were further digested in TrypLE solution into single-cell suspensions. Of note, BE or gastric mesenchymal cells were not isolated by this approach. The isolation of healthy stratified esophageal cells required a different extraction strategy (see below) (37). For the single-cell sequencing approaches, living DAPI^–^ cells were sorted into 384-well plates by FACS as previously described (54, 55).

Esophageal biopsies were digested for 30 min at 37 °C in 0.125% diluted trypsin solution (37). Freed, living, epithelial cells in the supernatant (DAPI^–^, EPCAM^+^) were sorted by FACS into 384-well plates for subsequent sequencing experiments (54, 55).

### Library Preparation for Single-Cell RNA and DNA Sequencing.

The scRNAseq library were prepared according to the CEL-seq2 protocol (55). The protocol for scDNAseq was developed by the van Oudenaarden laboratory (40). Proteins were digested by Proteinase K, genomic DNA by NLAIII, and the DNA fragments ligated to adaptors containing T7 polymerase binding sites. After pooling of cells, DNA was in vitro-transcribed and fragmented, and next-generation sequencing libraries were prepared and paired-end-sequenced on NextSeq500 (2 × 75 bp).

### Organoid Cultures and Clonal Expansion for WGS.

Single-cell suspensions were used to establish BE organoids in basement membrane extract and BE culture medium (Advanced DMEM/F12 supplemented with glutamine, Hepes and Pen/Strep, 20% R-spondin conditioned media, 1% Noggin, B27 with vitamin A, 10 mM nicotinamide, 0.5 nM WNT Surrogate, 50 µg/mL EGF, 500 nM A83-01, 10 nM prostaglandin E2, 1 µM SB 202190 inhibitor, and Primocin). Initially, Fungin was added to prevent fungal contamination during culture initiation. BE organoids were dissociated using TrypLE solution and split in a 1:5 ratio every 10 d. Of note, BE medium did not support the outgrowth of esophageal squamous or gastric organoids for long-term cultures, and BE organoids did not grow in gastric or esophageal culture medium (37).

For clonal expansion of organoids, single organoids were picked at passage 1, which were passaged separately. This step was repeated a second time to ensure clonality before expanding individual clones to isolate 1 μg genomic DNA (gDNA) using the ReliaPrep gDNA Tissue Miniprep System. As germline gDNA control, we pooled the mesenchymal leftover, after epithelial cell isolation, from all biopsies of the same patient. All samples were submitted to Macrogen for TruSeq PCR-Free library preparation and subsequent 30× WGS using the NovaSeq platform (2 × 150 bp).

### RNA In Situ Hybridization (RNAScope).

The staining was performed using RNAScope Multiplex Fluorescent Reagent Kit v2 (Advanced Cell Diagnostics) according to the manufacturer’s protocol (standard condition) (56). Images were acquired by a Leica SP8 confocal microscope. A list of the ordered probes is provided in Dataset S9.

### Data Analysis of WGS.

WGS data were mapped against human reference genome GRCh37 by using the BWA (v0.7.5) mapping tool (57) with settings 'bwa mem -c 100 -M.' Sequence reads were marked for duplicates by using Sambamba (v0.6.8) and realigned per donor by using Genome Analysis Toolkit (GATK) IndelRealigner (v3.8.1) Raw variants were multisample-called by using the GATK HaplotypeCaller (v3.8-0) (58) and GATK-Queue (v3.8-0) with default settings and additional option 'EMIT_ALL_CONFIDENT_SITES.' The quality of variant and reference positions was evaluated by using GATK VariantFiltration (v3.8-0) with options '-snpFilterName LowQualityDepth -snpFilterExpression “QD < 2.0” -snpFilterName MappingQuality -snpFilterExpression “MQ < 40.0” -snpFilterName StrandBias -snpFilterExpression “FS > 60.0” -snpFilterName HaplotypeScoreHigh -snpFilterExpression “HaplotypeScore > 13.0” -snpFilterName MQRankSumLow -snpFilterExpression “MQRankSum < −12.5” -snpFilterName ReadPosRankSumLow -snpFilterExpression “ReadPosRankSum < −8.0” -cluster 3 -window 35.' Full pipeline description and settings also available at https://github.com/UMCUGenetics/IAP/blob/develop/settings/UMCU_Genome_somatic.ini.

Single-nucleotide polymorphisms and INDELs were filtered based on the mapping quality score (MQ, >60) and a variant allele frequency (VAF >0.3) to exclude in vitro accumulated mutations (https://github.com/ToolsVanBox/SNVFI, https://github.com/ToolsVanBox/INDELFI). The distribution of variants was calculated and visualized using the R package MutationalPatterns (59).

### Data Analysis for scDNAseq.

Sequencing reads were mapped to the human genome using the NlaIII mapping pipeline of SingleCellMultiOmics package (see https://github.com/BuysDB/SingleCellMultiOmics/tree/master/singlecellmultiomics/snakemake_workflows/nlaIII). Copy number profiles were normalized by dividing by the median and multiplying by 2. Hierarchical clustering was performed and the amounts of clusters were manually defined for each patient. The breakpoints for each cluster were identified by using circular binary segmentation (see https://github.com/BuysDB/SingleCellMultiOmics/blob/master/singlecellmultiomics/bamProcessing/bamCopyNumber.py). The aneuploidy heat maps were generated in R using the pheatmap function and the cluster with hardly any copy number aberrations in each patient was defined as chromosomal stable.

Single-cell variants were called using BCFTOOLS 1.9-174, and since no patient matching germline controls was available all variants present in DBSNP (dbsnp_138.b37) were removed to filter against known germline variations. Additionally, variants overlapping with the CS cluster and variants which are shared between all patients were removed (*SI Appendix*, Fig. S4*B*). For each cluster, the enrichment of the remaining somatic variant was calculated using the Fisher exact test with a *P* value threshold of 0.05. The significantly enriched variants were exported to a VCF file. The trinucleotide patterns and cosine similarity plots were calculated using the R package MutationalPatterns (59). We focused our analysis on variants with a MQ score >20 and the SBS number was normalized to 100,000 reads per cell and a genome coverage of 1%. Of note, SBS calculation for scDNAseq data are noisier compared to WGS due to the lower sequencing depths (30× coverage in WGS and 0.5 to 1.0% coverage per cell in scDNAseq). The cluster-specific trinucleotide pattern was calculated by subtracting the frequencies observed in CS cells from the observed SBS frequencies in the other clusters. All negative values were set to 0. Reconstitution plots for SBS17a and SBS17b were calculated using the R package MutationalPatterns (59).

### Data Analysis for scRNAseq.

Sequencing reads were mapped to the human genome using the SingleCellMultiOmics pipeline (see https://github.com/BuysDB/SingleCellMultiOmics/tree/master/singlecellmultiomics/snakemake_workflows/cs2_scmo). The read counts tables were further analyzed by the RaceID3 algorithm (https://cran.r-project.org/web/packages/RaceID/index.html) (60). The data were filtered for cells >3,000 transcripts per cell and for genes that were expressed by at least three transcripts in at least one cell. Genes associated with clustering artifacts such as mitochondrial genes, MALAT1, and KCNQ1OT1 (37, 61, 62) were excluded from cluster calculation using the built-in FGenes or CGenes function. Cluster calculation was performed using the hclust method and outliers were identified using probthr = 2e-11 and outlg = 1. For the RaceID3 analysis using only genes in chromosomal stable regions, the same parameters were used but all genes located in CIN regions were excluded from the analysis using the FGenes function.

The built-in RaceID functions were used to calculate the differential gene expression (diffexpnb), the heat map (plotmarkergenes), t-SNE maps (plotexpmap), and the fraction dot plot (fractDotPlot).

## Supplementary Material

Supplementary File

Supplementary File

Supplementary File

Supplementary File

Supplementary File

Supplementary File

Supplementary File

Supplementary File

Supplementary File

Supplementary File

## Data Availability

Anonymized human sequencing data (scRNAseq, scDNAseq, and WGS) are available at the European Genome-Phenome Archive (EGA) under the accession number EGAS00001005221. Previously published data were used for this work (EGAS00001004695) (37).
