# Supplementary material for: Molecular characterization of Barrett’s esophagus at single-cell resolution
Source: Proc Natl Acad Sci U S A. 2021 Nov 18;118(47):e2113061118. doi: 10.1073/pnas.2113061118 (PMC8617519; doi:10.1073/pnas.2113061118)
Supplement: Supplementary File [file pnas.2113061118.sapp.pdf]

PNAS
www.pnas.org

Supplementary Information for
Molecular characterization of Barrett's esophagus at single-cell resolution

Georg A. Busslinger,
Buys de Barbanson,
Rurika Oka,
Bas L.A. Weusten,
Michiel de Maat,
Richard van Hillegersberg,
Lodewijk A.A. Brosens,
Ruben van Boxtel,
Alexander van Oudenaarden,
Hans Clevers

Correspondence:
Georg Busslinger (gbusslinger@cemm.oeaw.ac.at)
Hans Clevers (h.clevers@hubrecht.eu)

This PDF file includes:

Figures S1 to S9
Dataset S1 to S9

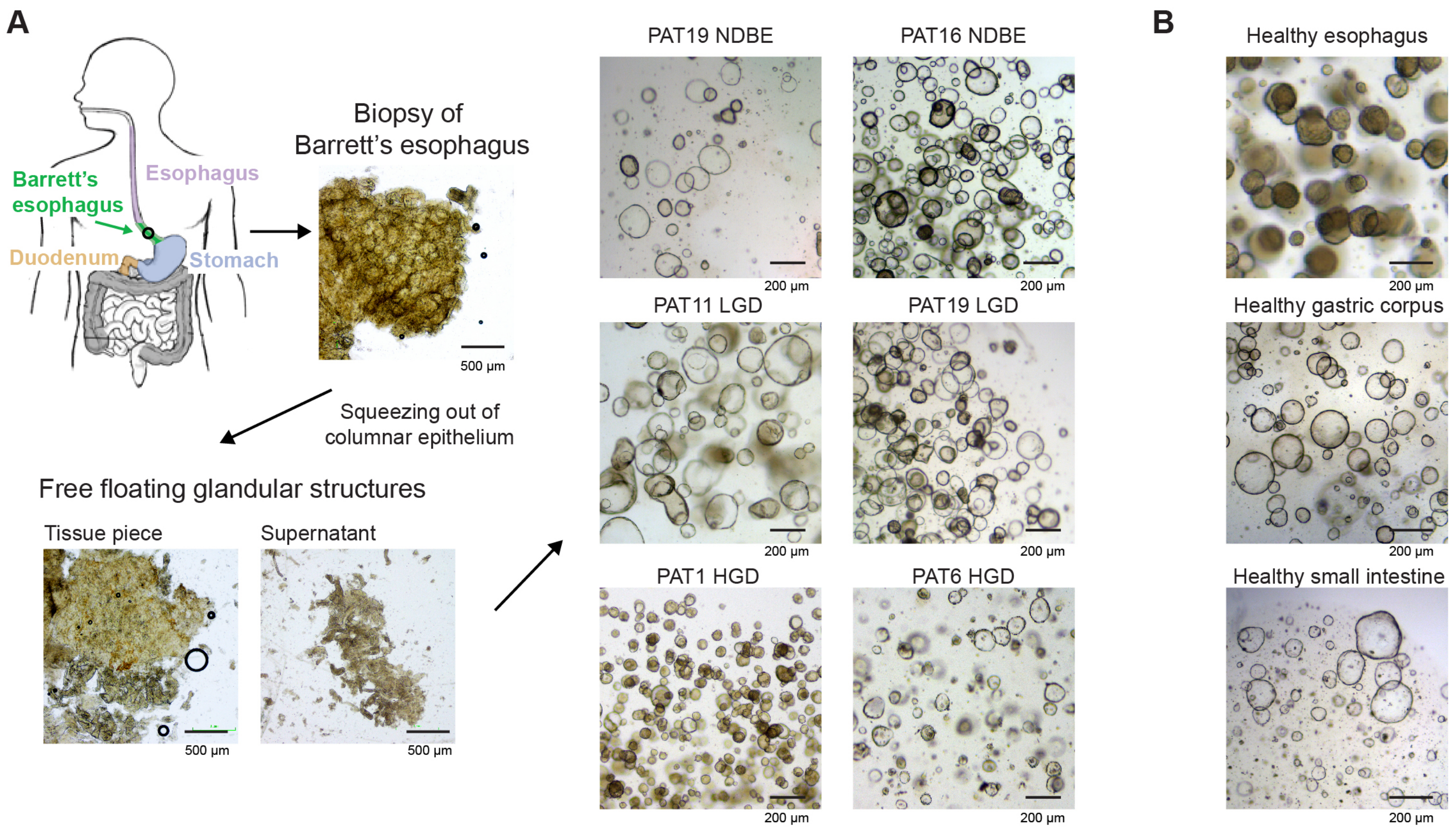

SI Appendix Figure S1. Generation of Barrett's esophagus organoids, related to Figure 1.
A. Schematic representation of the biopsy collection (top left) and the strategy for isolating columnar glands from BE biopsies (left). Isolated and dissociated columnar glands from different BE stages were embedded in basement membrane extract and in vitro propagated to give rise to organoid cultures (right).
B. Organoid cultures from matching non-diseased control tissue including esophagus (top), gastric corpus (middle) and small intestine (bottom).

A

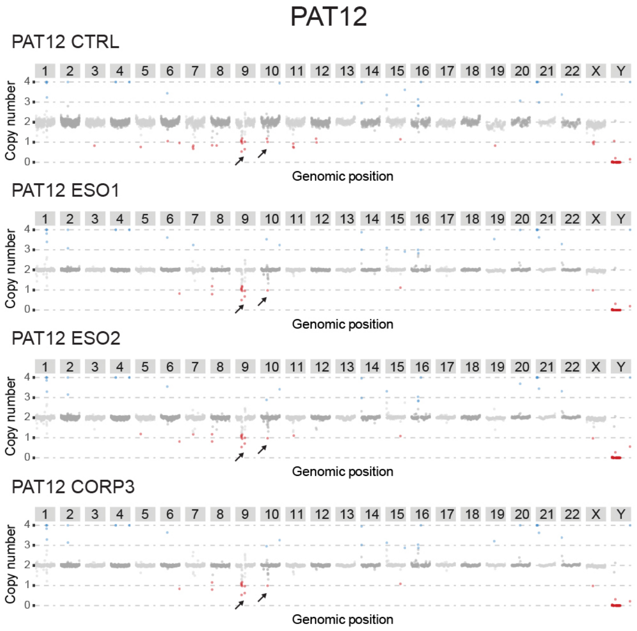

B

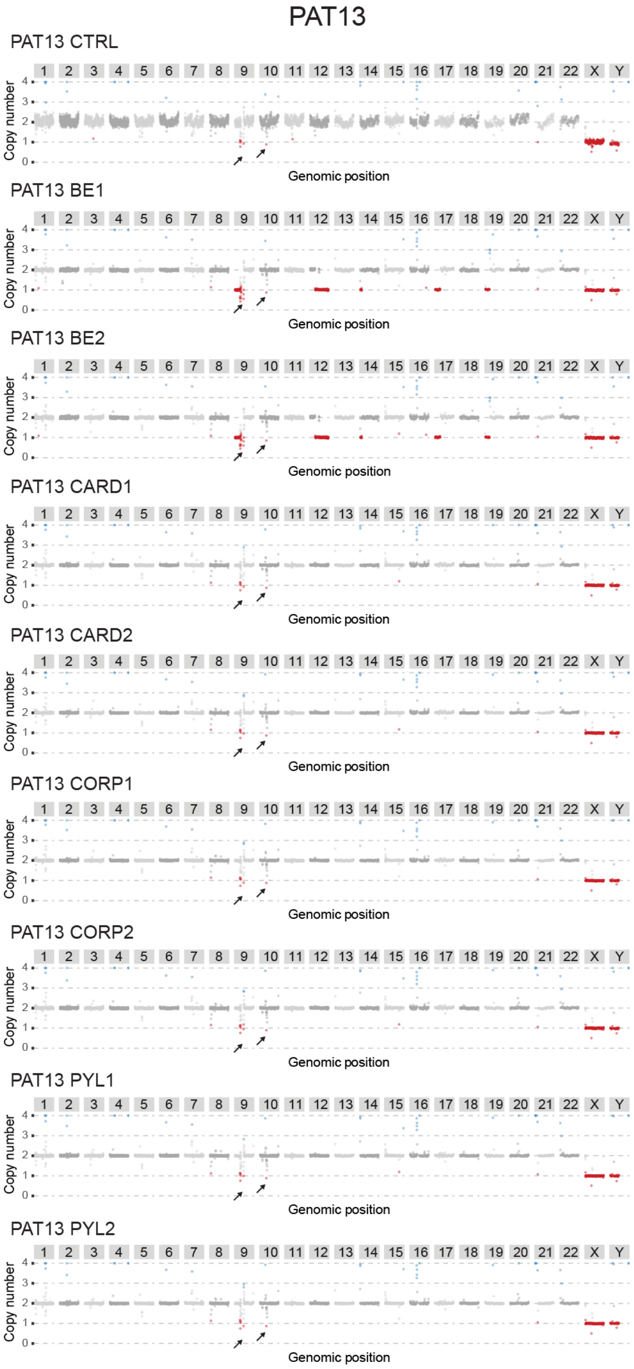

C

Significance of T>C and T>G distribution for anatomic regions (Fisher's test, P-values), related to Fig. 1D

	ESO	PYL	CORP	CARD
BE	7.53E-71	2.08E-59	8.53E-84	1.52E-67
CARD	0.50052	0.05162	0.05766	
CORP	0.00879	0.82535		
PYL	0.00890			

SI Appendix Figure S2. Karyotyping of the different organoid clones, related to Figure 1.

A,B. Plots depict the karyotypes determined for every analyzed clone including the control samples of PAT12 (**A**) and PAT13 (**B**). Black arrows indicate centromeric regions with low sequence coverage in samples even the controls. Due to the unreliable detection of such sequencing reads, these regions were not considered for the overall karyotype determination.

C. The P-values from Fisher's exact test calculations are shown for the distribution of T>C and T>G conversions between anatomic regions (rows versus columns).

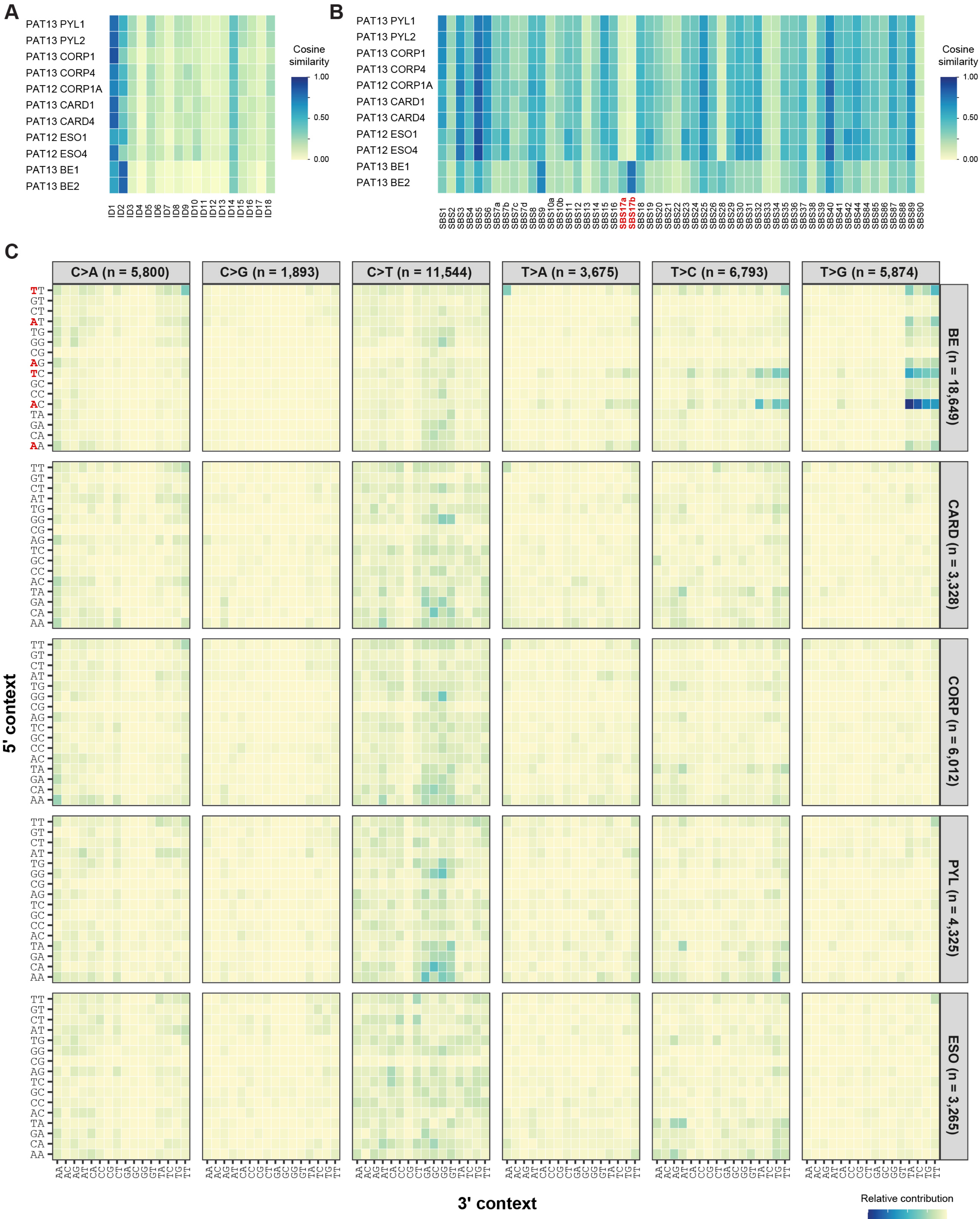

SI Appendix Figure S3. INDELs and SBS of organoid data, related to Figure 1.

A. Cosine similarities for COSMIC small insertion and deletion (ID) signatures.

B. Cosine similarities for COSMIC single-base substitution (SBS) signatures. The signatures SBS17a and SBS17b are highlighted in red. Please note that SBS9 and SBS17b share similar mutational features, which leads to the relative enrichment of SBS9 in cells with SBS17b patterns in the cosine similarity plot.

C. Overview of the relative contribution of different nucleotide conversions in the five-nucleotide context. Nucleotides enriched two nucleotides upstream of SBS17-specific T>C and T>G conversions are highlighted in red.

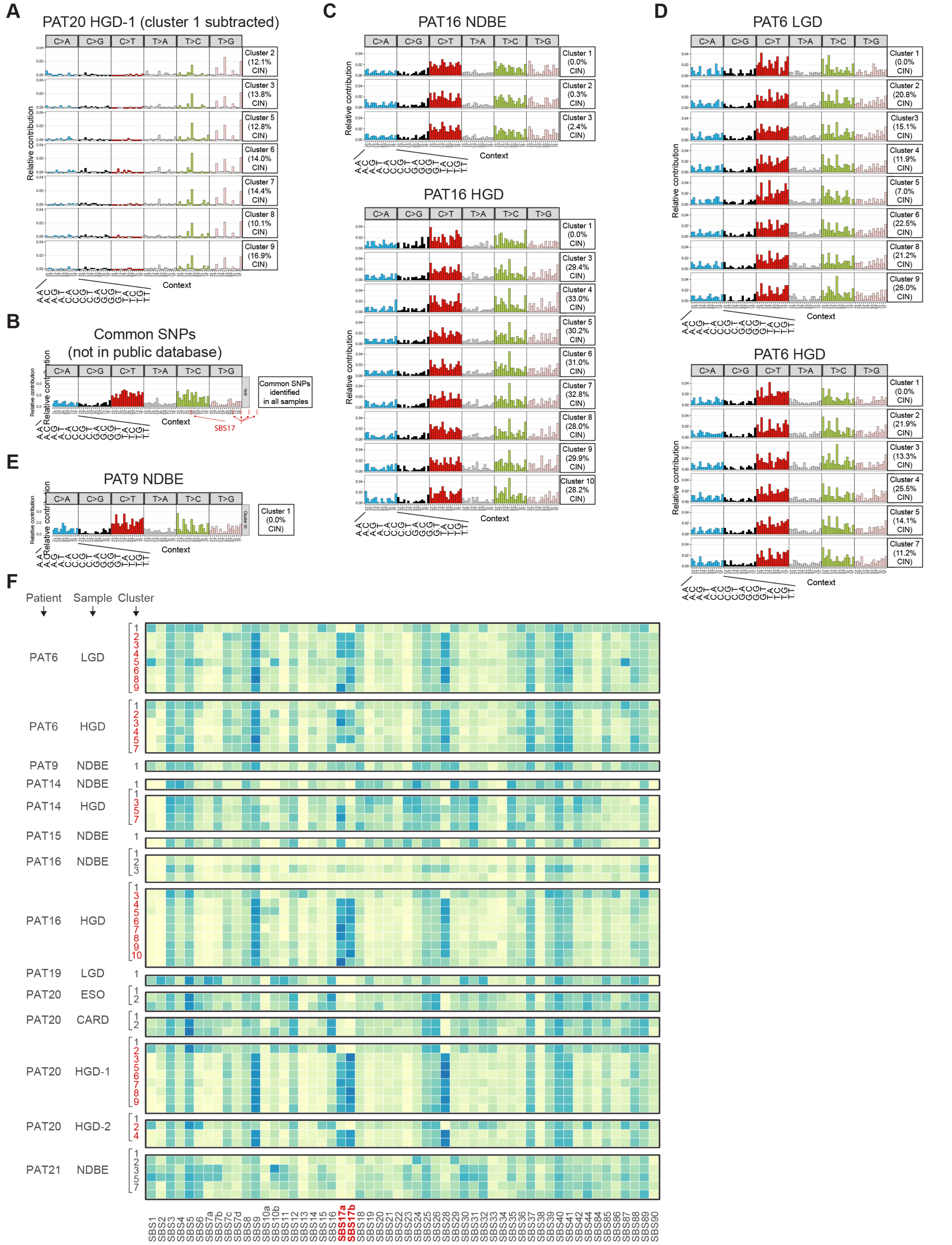

SI Appendix Figure S4. DNA alterations in BE biopsies, related to Figure 2 and 3.

A. Trinucleotide pattern for CIN cell cluster of PAT20 HGD-1 biopsy after subtracting the trinucleotide pattern of the respective CS cell cluster 1 (Fig. 2E).

B. Trinucleotide pattern of commonly detected SNPs in all scDNAseq sequenced samples, which were not part of the public available DBSNP database but were nonetheless considered as 'germline' SNPs. SBS17-specific nucleotide conversions are highlighted by red boxes.
C-E. Trinucleotide pattern of individual clusters for PAT16 (**C**), PAT6 (**D**) and PAT9 (**E**).
F. Cosine similarity to the COSMIC mutational signatures that was determined for all cell clusters of the different biopsies after subtracting the CS trinucleotide pattern, which was calculated based on all CS cell clusters of the different samples. The signatures SBS17a and SBS17b are highlighted in red as well as all CIN cell clusters (> 5% of chromosomal alterations). Please note that SBS9, SBS17b and SBS28 share similar features, which leads to the relative enrichment of SBS9 and SBS28 in cells with SBS17b patterns in the cosine similarity plot, particularly in cells with low sequencing coverage.

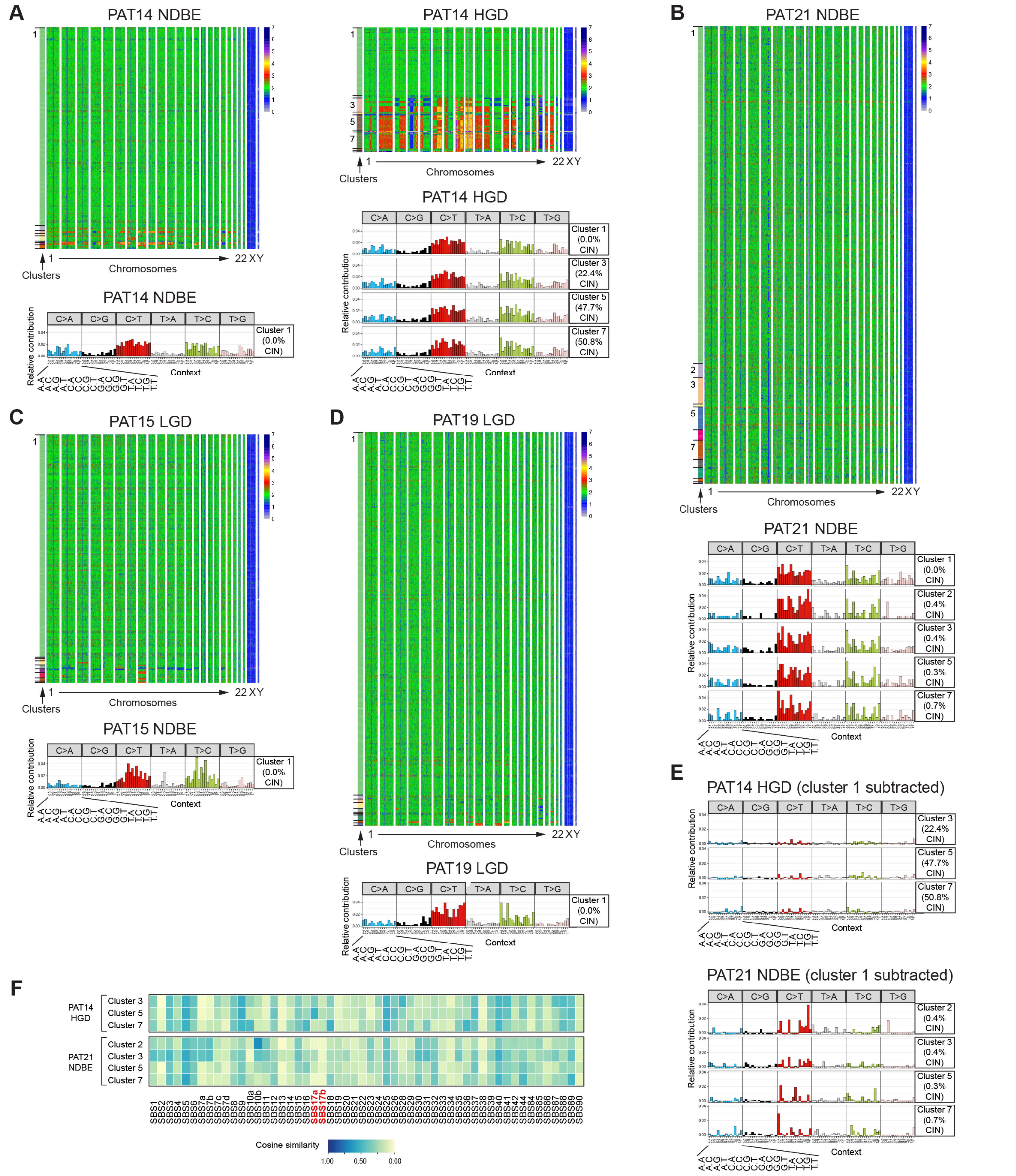

SI Appendix Figure S5. DNA alterations in BE biopsies of PAT14, PAT15, PAT19 and PAT21, related to Figure 2 and 3.

A-D. Heatmaps showing chromosomal stability at the single-cell level. The x-axis indicates individual chromosomes and the y-axis individual cells. Cell clusters are shown on the left (top). Overview of trinucleotide signatures for identified cell clusters in each sample (bottom).

E. Trinucleotide pattern of PAT14 HGD (top) and PAT21 NDBE (bottom) after subtracting the corresponding sample-internal CS cluster 1.

F. Cosine similarity of the PAT14 HDG and PAT21 NDBE samples to the COSMIC mutational signatures after subtracting the corresponding sample-internal CS cluster 1. The signatures SBS17a and SBS17b are highlighted in red.

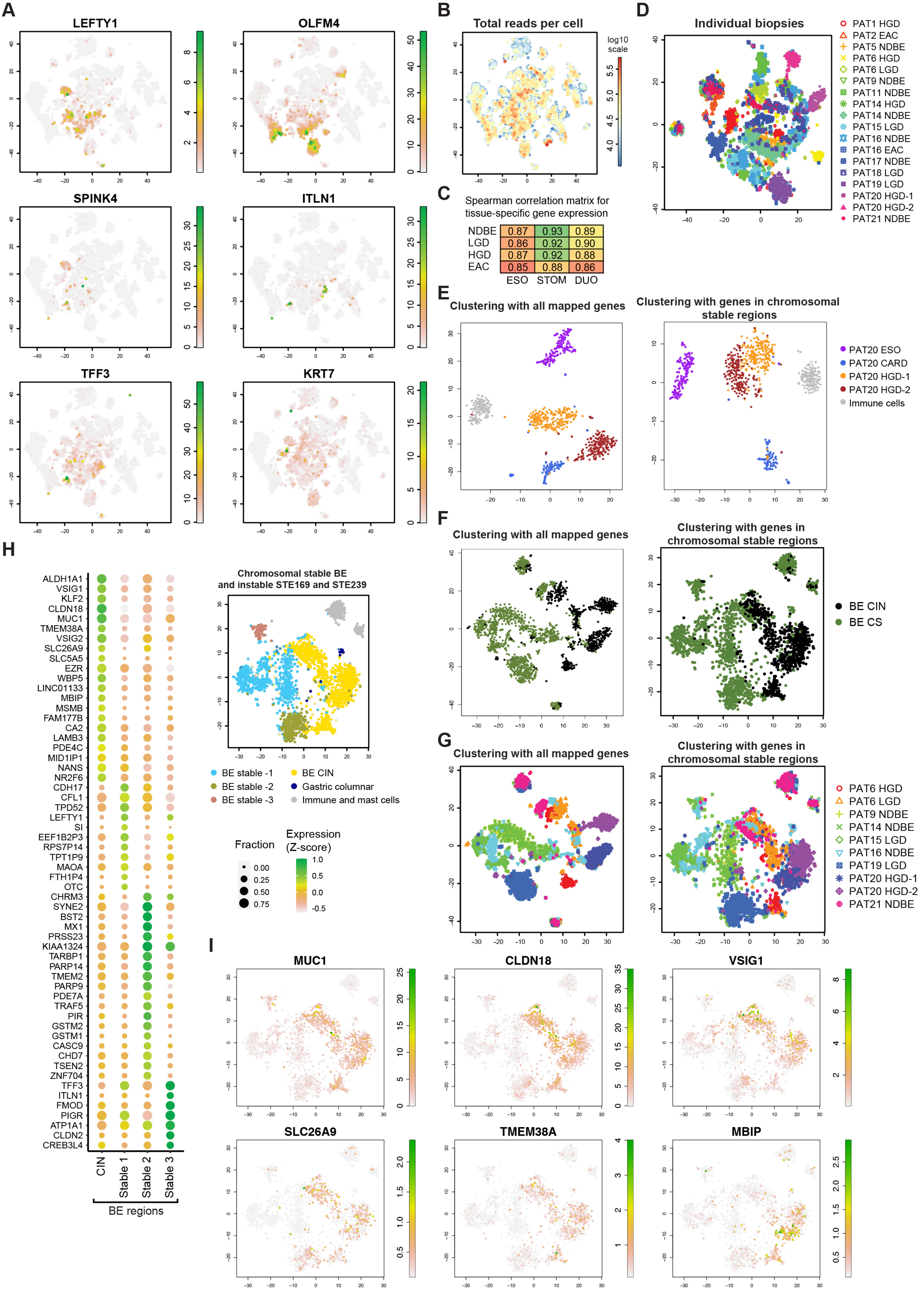

SI Appendix Figure S6

SI Appendix Figure S6. Analysis of scRNAseq data, related to Figure 4.

A. t-SNE maps showing the expression of LEFTY1, OLFM4, SPINK4, ITLN1, TFF3 and KRT7. The reference t-SNE map is shown in Fig. 5B.

B. t-SNE map showing total reads per cell. Data are depicted on a log10 scale and no bias is observed for cells with higher or lower read counts.

C. Spearman correlation matrix assessing the gene expression similarities between esophageal, gastric and duodenal cells with BE cells.

D. t-SNE maps displaying the scRNAseq data of only the BE samples. Cells were colored based on the pathology assessment (left) or based on the individual biopsies (right).

E. t-SNE maps displaying the scRNAseq data of PAT20. Cells were colored based on their anatomic regions. RaceID analysis using all genes (left) or only genes located in chromosomal stable regions (right).

F,G. t-SNE maps displaying the scRNAseq data, for which we had matching scDNAseq data. All NDBE samples were included in addition to the dysplastic samples from PAT6 and PAT20. Cells were colored based on the CIN data obtained by scDNAseq experiments (**F**) or based on the individual biopsies (**G**). RaceID analysis using all genes (left) or only genes located in chromosomal stable regions (right).

H. Gene expression analysis of CIN and CS stable cell clusters of BE, as shown by fraction dot plots. The different cell clusters are shown on the x-axis and enriched genes for CIN and CS regions are depicted on the y-axis. A schematic representation of the cell clusters is shown by a t-SNE map on the right. Each dot corresponds to the expression of one gene in one cell type. The dot size indicates the fraction of cells expressing a given gene within the indicated cell type, and the color denotes the mean expression level (scaled by Z-score transformation).

I. t-SNE maps showing the expression of MUC1, CLDN18, VSIG1, SLC26A9, TMEM38A and MBIP. The reference t-SNE map is shown in panel **F**.

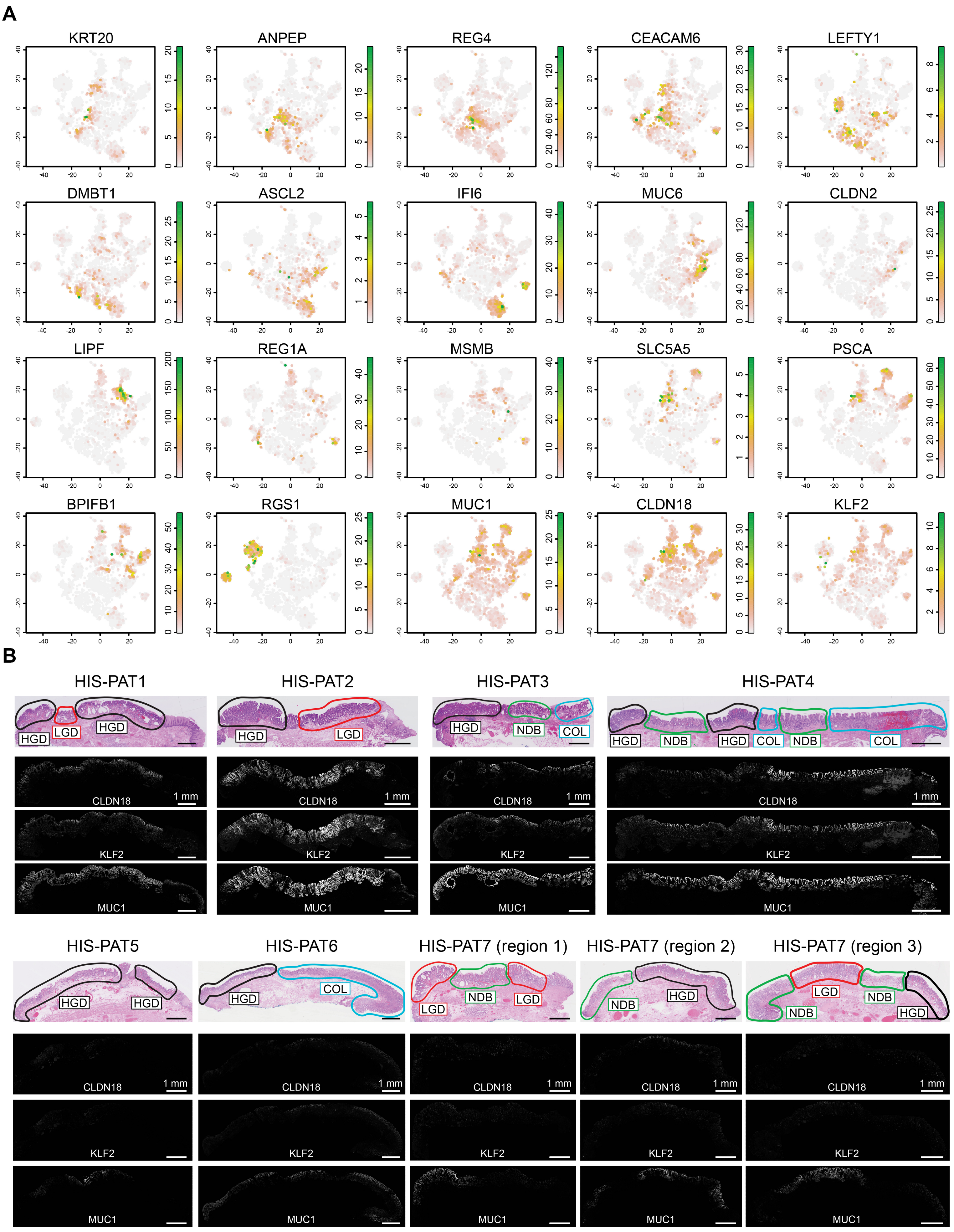

SI Appendix Figure S7

SI Appendix Figure S7. BE-specific gene expression and validation of CLDN18, KLF2 and MUC1 genes in histological resection specimen, related to Figure 4.

A. The specificity of the three selected marker genes, that showed preferential expression in CIN regions by scRNAseq, was analyzed by RNA in situ hybridization on nine endoscopic resection specimen containing different BE stages within the same histological sections. Scale bar: 1 mm.

B. t-SNE maps showing the expression of potentially stage-specific marker gene that localize to individual cell clusters. Together, they cover the entire spectrum of BE-specific cell clusters. The reference t-SNE map is shown in panel A.

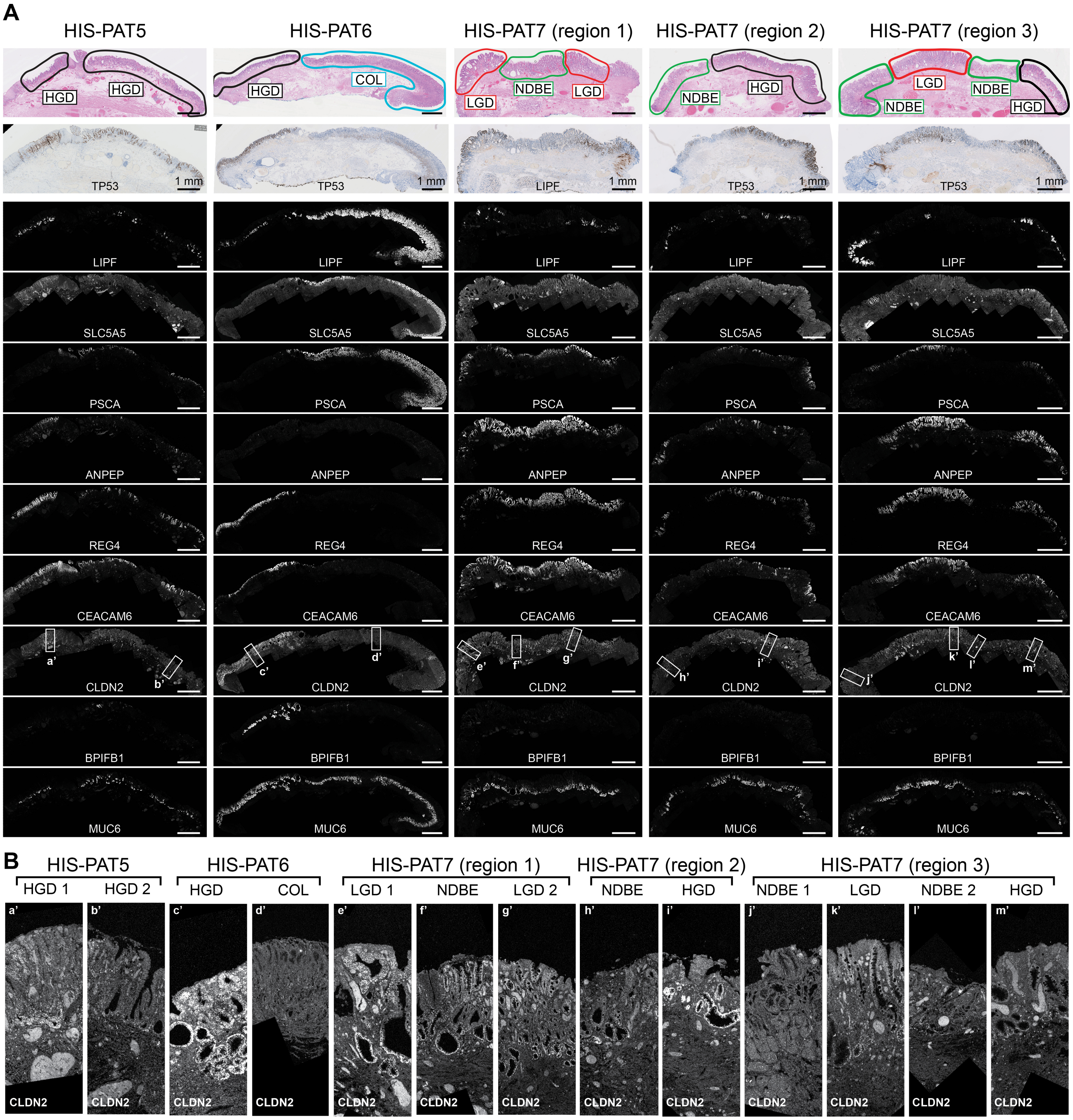

SI Appendix Figure S8. Candidate gene expression on histological resection specimen, related to Figure 5.

A. Five histological resection specimen containing different regions of BE were analyzed for the expression of LIPF, SLC5A5, PSCA, ANPEP, REG4, CEACAM6, CLDN2, BPIFB1 and MUC6 by RNA in situ hybridization. The first row shows the H&E staining including the pathological assessment of the BE stages, and the second row indicates the TP53 antibody staining. The NDBE regions of HIS-PAT7 sample (region 2 and 3) stained most likely negative for IM marker genes due to fixation artefacts that destroyed the surface epithelium for proper staining. Scale bar: 1 mm.

B. CLDN2 expression is shown at higher magnification for individual BE stages as indicated by rectangles in A.

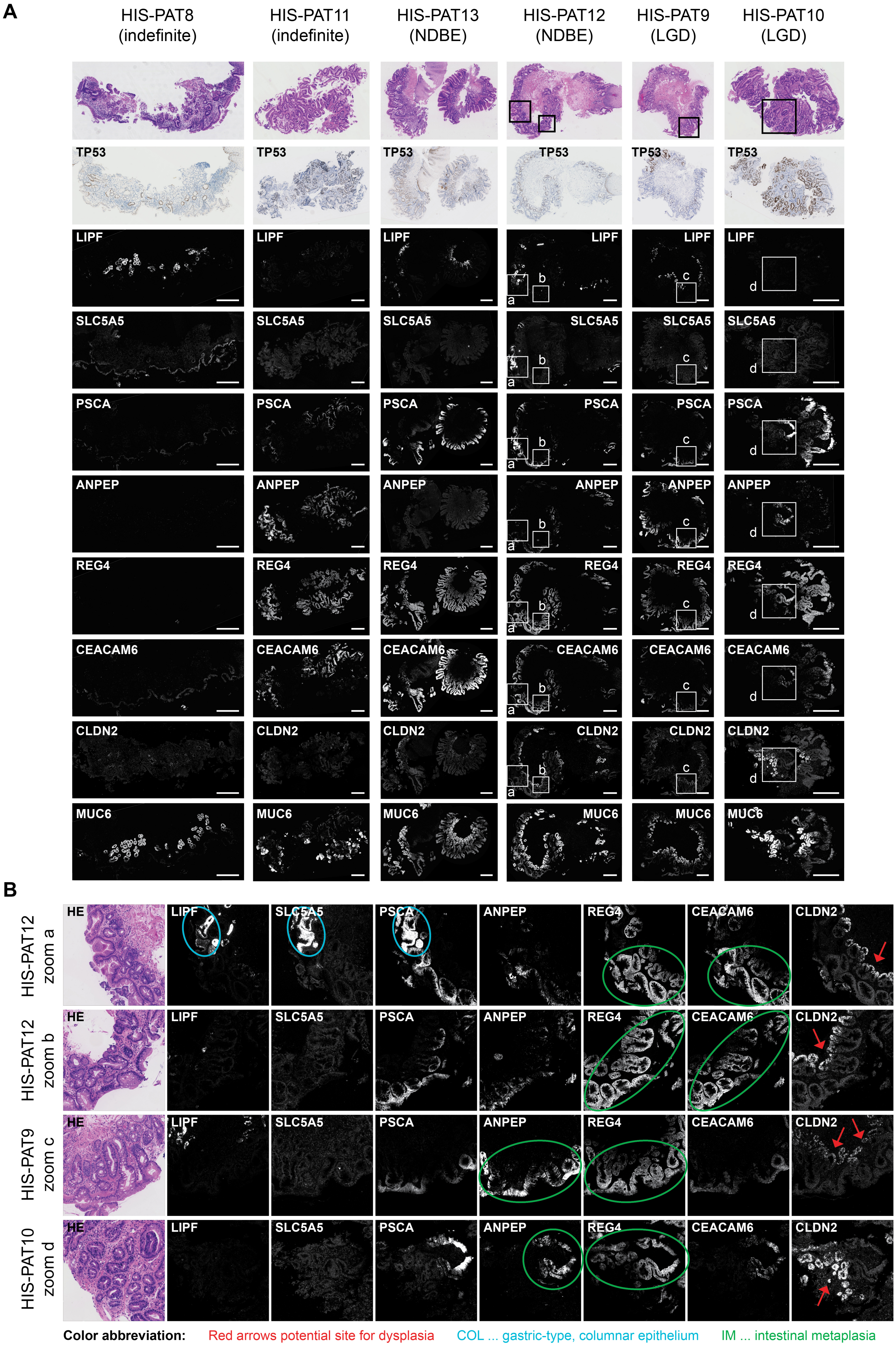

SI Appendix Figure S9

SI Appendix Figure S9. Histological confirmation of BE biopsies using COL and IM markers, related to Figure 5.

A. Histological analysis of BE biopsies from six patients, two ‘indefinite for dysplasia’, two non-dysplastic and two with LGD. H&E and TP53 antibody staining are on top followed by RNA in situ hybridization analysis of the expression of LIPF, SLC5A5, PSCA, ANPEP, REG4, CEACAM6, CLDN2 and MUC6. Scale bar: 300 µm.

B. Magnification of areas as indicated in **A**. The regions containing the columnar epithelium (COL) are marked by blue ovals and areas with IM by green ovals. Red arrows point to regions with increased CLDN2 expression.

Dataset S1. Patient information.

Overview providing information about the gender, age and pathology diagnosis of all patients included in this study.

Dataset S2. Overview of INDELs.

An overview is given for every organoid clone analyzed. On the left: overview about all detected INDELs; on the right: summary for double-nucleotide insertions.

Dataset S3. Single-base substitution overview.

An overview is given for the frequency of single-nucleotide substitutions for every organoid clone analyzed.

Dataset S4. Biopsy collection overview.

An overview is given for all collected biopsies from the 21 patients. Grey backgrounds mark the samples used for scRNAseq and orange those for scDNAseq. Samples with green boxes were only used to establish organoid cultures. The numbers indicate good quality cells used for either scRNAseq or scDNAseq experiments.

Dataset S5. Overview of the single-cell DNA sequencing clusters.

The number of bioinformatically identified clusters is listed, including their characteristics such as number of cells, reads and percentage of genome coverage per cluster. The last

three columns indicate the percentage of chromosome gains, loss and combined CIN normalized to the entire genome.

Dataset S6. Relative frequencies of immune and chromosomal stable cells in each biopsy.

The percentage of CS cells was calculated based on scDNAseq data, and the percentage of immune cells was determined by scRNAseq analysis of cells from the corresponding replica plates of the same biopsy.

Dataset S7. Single-cell RNA sequencing overview

The average number of transcripts and detected genes per cell are indicated for each analyzed biopsy.

Dataset S8. Gene names for heatmap plot in Fig. 4C

The tissue-specific genes, used for the heatmap calculation in Fig. 4C, are listed in the order they were used for plotting (top to bottom).

Dataset S9. Resources and reagents

Reagents and resources used in the method sections are listed.
